# The RBP2–BECN1–VEGFA axis orchestrates a self-amplifying circuit to drive malignant progression in gastric carcinoma

**DOI:** 10.3389/fonc.2026.1929764

**Published:** 2026-09-07

**Authors:** Panpan Tang, Ping Song, Xize Li, Yang Zou, Haocheng Wang, Chunyue Zheng, Shun Wang, Lin Lu, Jiping Zeng

**Affiliations:** 1 Department of Immunology, School of Basic Medicine, Qingdao University, Qingdao, Shandong, China; 2 Qingdao Central Hospital/School of Health and Life Sciences, University of Health and Rehabilitation Sciences, Qingdao, Shandong, China; 3 Department of Biochemistry and Molecular Biology, School of Basic Medical Sciences, Cheeloo College of Medicine, Shandong University, Jinan, Shandong, China; 4 Department of Surgical Oncology, Affiliated Hangzhou First People’s Hospital, Westlake University School of Medicine, Hangzhou, Zhejiang, China; 5 Shanghai Sixth People’s Hospital affiliated to Shanghai Jiao Tong University School of Medicine, Shanghai, China

**Keywords:** angiogenesis, autophagy, gastric carcinoma, RBP2, signal transduction

## Abstract

**Objective:**

Gastric carcinoma (GC) is a leading cause of cancer-related mortality, often driven by epigenetic dysregulation. This study investigates the role of Retinoblastoma-binding protein 2 (RBP2), a chromatin-associated transcriptional regulator frequently overexpressed in GC. We specifically focus on elucidating how RBP2 functions as a transcriptional regulator​ within the autophagy-angiogenesis network and its impact on disease progression.

**Methods:**

The regulatory axis was dissected using integrated approaches, including transcriptomic profiling, cellular and molecular biology assays, and *in vivo* animal models. Clinical specimen analysis was performed to correlate RBP2 expression with autophagy and angiogenesis markers. Functional validation involved gain- and loss-of-function experiments to delineate the signaling circuitry linking RBP2 to BECN1 and VEGFA.

**Results:**

We discovered that RBP2 drives GC progression by establishing an autophagy-angiogenesis positive feedback loop. RBP2 transcriptionally regulates BECN1 through promoter-dependent mechanisms supported by dual-luciferase reporter and ChIP-qPCR assays, thereby inducing protective autophagy. This autophagy enhances VEGFA secretion, which upregulates RBP2 via the inactivation of the JNK/p53/miR-212 axis, thereby relieving post-transcriptional repression. This self-reinforcing circuit facilitates sustained gastric carcinogenesis.

**Conclusion:**

Our findings support a novel “RBP2-BECN1 -VEGFA-JNK/p53/miR-212-RBP2” regulatory axis that links epigenetic regulation to autophagic survival and angiogenic signaling. This study elucidates a key mechanism underlying GC pathogenesis and suggests potential targets for combinatorial therapeutic strategies.

## 1 Introduction

GC remains a highly lethal malignancy, with an estimated 968,000 new cases and 659,800 deaths annually according to the GLOBOCAN 2022 database (1–3). Despite advancements in multimodal therapies, the prognosis for advanced GC remains dismal, with 5-year survival rates below 20%, largely due to tumor recurrence, metastasis, and therapeutic resistance (4–8). Elucidating the molecular mechanisms driving GC progression is therefore critical for identifying effective therapeutic targets.

Within the tumor microenvironment (TME), autophagy and angiogenesis represent two interconnected hallmarks of cancer. While basal autophagy suppresses tumor initiation, established tumors hijack this process to survive metabolic stress, such as hypoxia and nutrient deprivation (9, 10). Concomitantly, autophagy promotes VEGFA secretion and thus mediates angiogenesis, creating a vicious cycle that fuels tumor growth (11–18). However, the precise epigenetic regulators governing this interplay remain elusive.

GC also involves profound dysregulation of gastric epithelial identity genes. For instance, the gastric-specific cytoprotective factor Gastrokine 2 (GKN2) is frequently downregulated in GC, and its expression correlates with the transcription factor FOXA2, which establishes chromatin accessibility at specific enhancers (19, 20). Such epigenetic mechanisms where transcription factors and chromatin modifiers cooperate to reshape cell identity highlight the broader theme of epigenetic dysregulation in GC, setting the stage for investigating how chromatin regulators like RBP2 may rewire transcriptional programs to drive malignancy.

The histone demethylase RBP2 (also known as KDM5A) is characterized by its catalytic domain responsible for the removal of H3K4me2/3 marks (21, 22). Consistent with its context-dependent roles, RBP2 has been implicated in diverse oncogenic processes. It confers chemoresistance in non-small cell lung cancer (23), hyperactivates the PI3K/AKT signaling pathway in prostate cancer (24), arrests differentiation and sustains stemness in acute myeloid leukemia (25), and promotes epithelial-mesenchymal transition in hepatocellular carcinoma (22). Beyond its canonical enzymatic function, RBP2 possesses intrinsic sequence-specific DNA-binding capacity and acts as a transcriptional regulator in various malignancies (22, 26). Notably, rather than functioning solely as a transcriptional repressor, RBP2 can also serve as a direct transcriptional activator (22, 27). In line with this, we previously demonstrated that RBP2 directly occupies the *VEGFA* promoter to drive its transcription and promote angiogenesis in GC (28). Intriguingly, bioinformatics analysis revealed putative RBP2 binding motifs within the *BECN1* promoter, a master regulator of autophagy. Given the critical crosstalk between autophagy and angiogenesis in the heterogeneous TME (17), and building on our prior evidence of RBP2’s role as a transcriptional activator on VEGFA expression, we hypothesized that RBP2 functions as a transcriptional integrator linking these two processes via a direct regulatory mechanism.

Building on these premises, this study aimed to elucidate whether RBP2 functions as a transcriptional regulator bridging autophagy and angiogenesis. We sought to determine whether RBP2 establishes a self-amplifying epigenetic loop that sustains tumor progression. Deciphering how this chromatin-associated regulator orchestrates the autophagy-angiogenesis network provides mechanistic insights into GC pathogenesis and highlights potential combinatorial therapeutic strategies.

## 2 Materials and methods

### 2.1 Clinical tissue specimens

A total of 36 paired GC tissues and adjacent normal tissues were retrospectively collected from Qilu Hospital of Shandong University in 2020. All patients were histopathologically diagnosed with primary GC and had not received neoadjuvant therapy. This study was approved by the Ethics Committee of Shandong University School of Medicine (No. ECSBMSSDU2020-1-017). Tissues were snap-frozen in liquid nitrogen immediately after resection and stored at ‐80 °C until use.

### 2.2 Cell culture

Human GC cell lines (AGS and HGC-27) were obtained from the Cell Bank of the Chinese Academy of Sciences (Shanghai, China), where they were authenticated by short tandem repeat (STR) profiling. AGS cells were maintained in Ham’s F12 medium supplemented with 12% fetal bovine serum (FBS), while HGC-27cells were cultured in RPMI-1640 medium with 10% FBS. All cells were incubated at 37 °C in a humidified atmosphere containing 5% CO_2_. Cells were routinely tested for mycoplasma contamination using the MycoAlert™ Mycoplasma Detection Kit (Lonza, Switzerland) and confirmed to be mycoplasma-free before use in experiments.

### 2.3 Cell transfection

Plasmid transfection was performed using Hieff Trans^®^ Liposomal Transfection Reagent (Yeasen, China) when cells reached 80%–90% confluence. Small interfering RNAs (siRNAs) transfection was conducted with Lipofectamine 2000 (Invitrogen, USA) at a cell density of 30%–40%. The RBP2 overexpression plasmid was gifted by Professor Xu’s laboratory and preserved in our laboratory. P53 plasmid, PGL-3-basic vector, and TK renilla plasmid were stored in our laboratory. The *BECN1* promoter luciferase reporter plasmid was constructed via molecular cloning in this study.

Gene-specific siRNAs targeting the human genes *RBP2*, *BECN1*, and *VEGFA*, and negative control siRNA (si-NC), were synthesized by Sangon Biotech (Shanghai, China). The siRNA sequences were as follows: si-NC: 5′-UUCUCCGAACGUGUCACGUTT-3′; si-VEGFA: 5′-GAGCGGAGAAAGCAUUUGUTT-3′; si-BECN1: 5′-GCUGCCGUUAUACUGUUCUTT-3′; si-RBP2: 5′-CCAGCACCACCUCCUUCCUUCAUAA-3′.

### 2.4 Reverse transcription - quantitative real-time PCR

Total RNA was isolated using TRIzol reagent (Invitrogen). For mRNA detection, reverse transcription was performed using an AG-11707 kit (Agbio, China). For miR-212​ detection, specific reverse transcription primers (Agbio) were used. RT-qPCR was conducted on a Bio-Rad CFX96 system. Relative expression was normalized to GAPDH (for mRNA) or U6 (for miRNA) and calculated using the 2^−Δct^ method. Primer sequences are listed in **Supplementary Table 1**.

### 2.5 Colony formation assay

Transfected GC cells were resuspended and counted, with 800 cells seeded into each well of 6-well plates (3 replicate wells per group). After 14 days of continuous culture, cells were fixed with methanol for 8 min and stained with 0.1% crystal violet solution for 45 min. After rinsing and air drying, cell colonies containing more than 50 cells were counted and photographed for statistical analysis.

### 2.6 Western blot analysis

Total cellular protein was extracted using RIPA lysis buffer (Beyotime, China) supplemented with 1% PMSF protease inhibitor (Beyotime). Protein concentration was quantified via the BCA protein assay kit (Beyotime). Equal amounts of protein samples were separated by SDS-PAGE electrophoresis and transferred to PVDF membranes (Merck Millipore, Germany). Membranes were blocked with 5% skimmed milk at room temperature for 1.5 h and incubated with primary antibodies overnight at 4 °C. Primary antibodies included anti-RBP2 (1:5000, Abcam, UK, ab194286), anti-BECN1(1:3000, Santa Cruz, USA, sc-48341), anti-VEGFA (1:1000, Abcam,UK, ab46154), anti-p53 (1:1000, Santa Cruz,USA, sc-6243), anti-JNK (1:1000, CST, USA, #9252), anti-p-JNK (1:1000, CST, USA, #4668), anti-GAPDH (1:1000, CST, USA, #2118), and anti-Tubulin (1:1000, CST, USA, #2128). After washing with TBST buffer, membranes were incubated with HRP-conjugated secondary antibodies (1:5000, ZSGB-BIO, China) at room temperature for 1 h. Protein bands were visualized using enhanced chemiluminescence (ECL) reagent (Millipore, USA). GAPDH or Tubulin was used as the internal loading control. Band gray values were quantified using ImageJ software.

### 2.7 Bioinformatic prediction of RBP2-binding motifs

Prediction of RBP2-binding motifs in the BECN1 promoter was performed using the JASPAR 2024 database (matrix ID: MA0764.1 for KDM5A) and the HOCOMOCO v11 database (KDM5A motif, H12MOCOMOCOv11core). The BECN1 promoter region (-1972 bp to +497 bp relative to the Transcription Start Site [TSS at chr17: 42824282], GRCh38/hg38, chr17: 42,823,785 – 42,826,254) was scanned for matches to the canonical CCGCCC recognition sequence (26). Three high-confidence motifs were identified at positions −574/−569 (motif 1), −261/−256 (motif 2), and −205/−200 (motif 3) relative to the TSS (**Supplementary Table 2**). All three motifs were included in the luciferase reporter construct (spanning -1500 to +200 bp of the BECN1 promoter).

### 2.8 Dual-luciferase reporter assay

AGS and HGC-27 cells were seeded into 24-well plates at a density of 1×10^5^ cells per well. For overexpression assays, cells were co-transfected with RBP2 overexpression plasmid, TK renilla plasmid, and PGL-3-*BECN1* promoter plasmid. For knockdown assays, cells were transfected with RBP2 siRNA for 24 h, followed by co-transfection with TK and *BECN1* promoter plasmids. After 48 h of transfection, cells were lysed with PBL lysis buffer. Dual-luciferase activity was detected using the Promega E1941 detection kit. Firefly luciferase activity was normalized to renilla luciferase activity for statistical analysis.

### 2.9 Chromatin immunoprecipitation-qPCR assay

ChIP assays were performed using the SimpleChIP^®^ Enzymatic Chromatin IP Kit (Cell Signaling Technology, #9003) with minor modifications. Briefly, AGS cells stably overexpressing RBP2, stably depleted of RBP2, and their respective control cells were seeded into 10-cm dishes at a density of 2×10^6^ cells per dish. When reaching 80–90% confluence, cells were cross-linked with 1% formaldehyde for 10 min at room temperature and quenched with 125 mM glycine for 5 min. Cells were harvested and nuclei were isolated, followed by chromatin digestion with micrococcal nuclease. After enzymatic digestion, samples were further sonicated to ensure optimal chromatin fragmentation. For each immunoprecipitation, 10 μg of digested chromatin was incubated with 2 μg of anti-RBP2 antibody (Abcam, ab70892), anti-H3K4me3 antibody (Abcam, ab8580), anti-histone H3 antibody (CST, #4620, as input control for histone modifications), or normal rabbit IgG (CST, #2729) overnight at 4 °C with rotation, followed by precipitation with Protein G magnetic beads for 2 h. Beads were washed sequentially with low-salt, high-salt, LiCl, and TE buffers. Cross-links were reversed by incubation at 65 °C overnight, and DNA was purified after proteinase K digestion. qPCR was performed using primers targeting the three predicted RBP2-binding sites in the BECN1 promoter. Primer sequences are listed in **Supplementary Table 1**. Enrichment was calculated as the percentage of input DNA and normalized to IgG control for statistical analysis.

### 2.10 Xenograft models

Six-week-old male BALB/c nude mice were housed under specific pathogen-free conditions. HGC-27 cells (5 × 10^6^) suspended in 100 μL PBS were subcutaneously injected into the right flanks. Tumor volumes were measured every 2 days using calipers and calculated as V = 0.5 × length × width². Mice were euthanized on day 18-21, and tumors were excised for analysis. Mice were euthanized by cervical dislocation under deep anesthesia (pentobarbital sodium 50 mg/kg), with anesthesia depth confirmed by loss of pedal withdrawal reflex, following the AVMA Euthanasia Guidelines (2020). All procedures were approved by the Animal Ethics Committee of Shandong University (No. ECSBMSSDU2020-2-029) and complied with the Guide for the Care and Use of Laboratory Animals and the ARRIVE guidelines.

### 2.11 Immunohistochemistry staining

Paraffin-embedded tissue sections were deparaffinized, rehydrated, and subjected to antigen retrieval. After blocking endogenous peroxidase activity and non-specific binding, sections were incubated with primary antibodies against RBP2 (1:300, Sigma, USA, sab4301220), BECN1 (1:500, Santa Cruz, USA, sc-48341), and VEGFA (1:250, Abcam, UK, ab32152) overnight at 4 °C. After PBS washing, sections were incubated with HRP-conjugated secondary antibodies at room temperature for 1 h. DAB staining and hematoxylin counterstaining were performed following the kit instructions. Finally, sections were dehydrated, cleared, and mounted for microscopic observation and quantitative analysis.

### 2.12 Immunofluorescence staining

GC cells were seeded on cell slides and cultured for 24 h. Cells were fixed with 4% paraformaldehyde for 15 min, permeabilized with 0.3% Triton X-100 for 15 min, and blocked with 5% BSA for 1 h. Subsequently, cells were incubated with anti-LC3A/B primary antibody (1:200, Santa Cruz, sc-398822) overnight at 4 °C. After PBST washing, cells were incubated with fluorescent secondary antibody (1:100, Proteintech, USA, SA00013-3) in the dark for 1 h. Nuclei were stained with DAPI, and slides were sealed with anti-fluorescence quenching agent. LC3 puncta formation was observed and photographed via fluorescence microscopy.

### 2.13 Transwell invasion assay

Transfected GC cells (5×10^4^ cells) were seeded into the upper chamber of Matrigel-precoated Transwell inserts (Corning, USA). The lower chamber was supplemented with complete medium containing 10%–12% FBS. After 48 h of culture, invasive cells in the lower chamber were fixed with methanol and stained with crystal violet. Invasive cells were photographed and counted under an optical microscope to evaluate cell invasive capacity.

### 2.14 Transmission electron microscopy analysis

HGC-27 cells with stable RBP2 overexpression and control cells were collected. TEM sample preparation, including fixation with 2.5% glutaraldehyde and 1% osmium tetroxide, gradient dehydration, epoxy resin embedding, ultrathin sectioning, and double staining with uranyl acetate and lead citrate, was performed by Electron Microscopy Laboratory, Clinical Medical Research Center, Affiliated Hospital of Jining Medical University. Autophagosome morphology and quantity were analyzed using a FEI Talos L120C 120KV TEM (Thermo Fisher Scientific, USA) across multiple random fields.

### 2.15 Statistical analysis

All experiments were repeated at least three times independently. Experimental data were presented as mean ± standard deviation (SD). GraphPad Prism 8.0.2 software was used for statistical analysis and graph plotting. Student’s t-test was used for two-group comparison, and one-way ANOVA was applied for multiple-group comparison. *P* < 0.05 was considered statistically significant.

## 3 Results

### 3.1 RBP2 transcriptionally regulates *BECN1* to induce autophagy in GC

**Figure 1:**
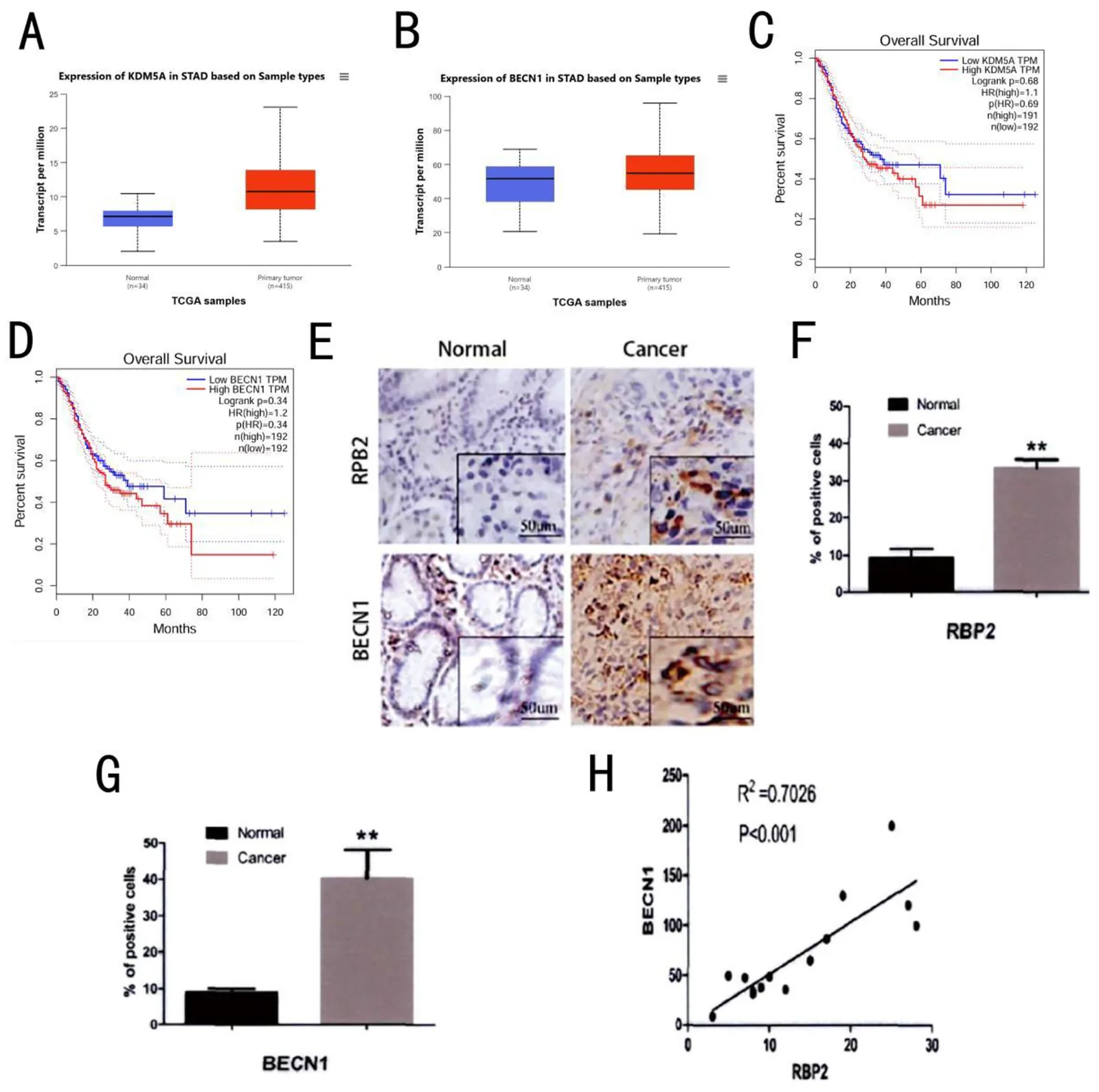
RBP2 positively correlates with BECN1 in GC. **(A-D)** Analysis of RBP2 and BECN1 expression in GC tissues versus normal tissues using the UALCAN-TCGA database. **(E)** Representative IHC staining of RBP2 and BECN1 in 21 paired GC specimens. Scale bar, 50 μm. **(F, G)** Quantitative analysis of RBP2 and BECN1 positivity rates. **(H)** Pearson correlation analysis of RBP2 and BECN1 protein levels (R = 0.7026, *P* < 0.001). Data are mean ± SD. **P < 0.01.

Given our previous observation that RBP2 directly transactivates VEGFA to promote angiogenesis (28), we hypothesized that RBP2 might coordinate autophagic and angiogenic programs via a transcriptional cascade. To test this, we first examined the relationship between RBP2 and the core autophagy gene *BECN1*. Analysis of the UALCAN-TCGA dataset revealed that both RBP2 and BECN1 are significantly upregulated in GC tissues compared to adjacent normal tissues, and high expression correlates with poor patient survival (**Figures 1A–D**). Immunohistochemistry (IHC) staining of 21 paired clinical specimens confirmed elevated RBP2 and BECN1 protein levels in tumors, with a strong positive correlation (*R* = 0.7026, *P* < 0.001) (**Figures 1E–H**).

**Figure 2:**
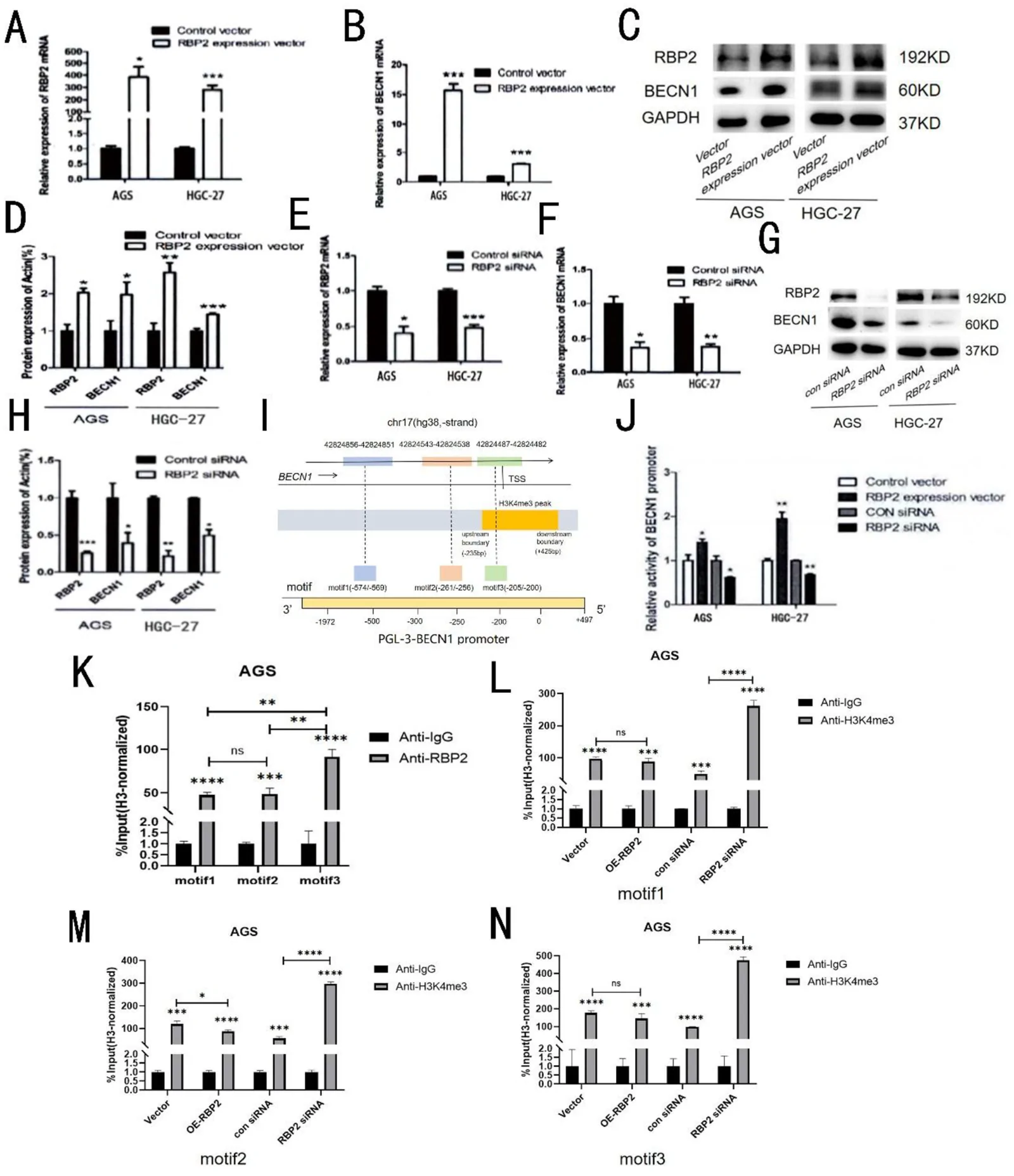
RBP2 transcriptionally regulates *BECN1* expression in GC. **(A-H)** Effects of RBP2 overexpression **(A-D)** or knockdown **(E-H)** on BECN1 mRNA and protein levels in AGS and HGC-27 cells. **(I)** Schematic diagram of three predicted RBP2-binding motifs, the BECN1 promoter luciferase reporter construct, and ChIP-qPCR primer design. The genomic coordinates on chromosome 17 (hg38, -strand) are shown at the top, and the binding sites include site 1: 42824851-42824856, site 2: 42824538-42824543, and site 3: 42824482-42824487. The TSS and H3K4me3-enriched region are indicated in the middle. Three high-confidence RBP2-binding motifs are located at positions −574/−569, −261/−256, and −205/−200 relative to the TSS. The BECN1 promoter luciferase reporter construct is shown at the bottom, with blue arrows indicating the three ChIP-qPCR primer pairs designed to amplify regions corresponding to the RBP2-binding motifs. **(J)** Dual-luciferase reporter assay showed that RBP2 enhanced *BECN1* promoter activity. **(K)** ChIP-qPCR assays showing RBP2 enrichment at the three predicted binding sites in the BECN1 promoter. **(L-N)** ChIP-qPCR assays showing H3K4me3 levels at the three BECN1 promoter sites upon RBP2 overexpression or knockdown. Data are mean ± SD. *P < 0.05, **P < 0.01, ***P < 0.001, ****P < 0.0001.

*In vitro*, RBP2 overexpression upregulated *BECN1* mRNA and protein levels in both AGS and HGC-27 cells, whereas RBP2 knockdown produced the opposite effect (**Figures 2A–H**). To determine whether RBP2 regulates BECN1 at the transcriptional level, we constructed a luciferase reporter containing the *BECN1* promoter region (-1972 to +497 bp relative to the TSS), which includes three predicted RBP2-binding motifs (CCGCCC, the canonical RBP2 recognition sequence). Dual-luciferase assays showed that RBP2 overexpression enhanced BECN1 promoter activity by approximately 2.1-fold, while its depletion suppressed it by ~40% (**Figures 2I, J**). To further confirm whether RBP2 physically occupies the BECN1 promoter, we performed ChIP-qPCR assays in AGS cells using an anti-RBP2 antibody. Our results revealed significant enrichment of RBP2 at all three predicted binding sites compared to IgG control. Among the three sites, motif 3 (−205/−200, the most proximal to the TSS, located within the H3K4me3 peak) showed the strongest RBP2 enrichment, followed by motif 2 (−261/−256, at the upstream boundary of the H3K4me3 peak) and motif 1 (−574/−569, distal to the H3K4me3 peak). Quantitative comparison confirmed that motif 3 enrichment was significantly higher than both motif 1 (1.92-fold, *P* < 0.01) and motif 2 (1.87-fold, *P* < 0.01), while motif 2 showed only a slight, non-significant increase compared to motif 1 (1.03-fold, *P*>0.05) (**Figure 2K**, **Supplementary Figure 1A**).

We next examined whether RBP2 exerts its demethylase activity at the BECN1 promoter by measuring H3K4me3 levels at the same three sites in AGS cells stably overexpressing RBP2, stably depleted of RBP2, and their respective control cells (**Supplementary Figures 1B, C**). Consistent with the known function of RBP2 as an H3K4me2/3 demethylase, RBP2 overexpression decreased H3K4me3 levels at all three sites, with the most pronounced and statistically significant reduction observed at motif 2 (*P* < 0.05). Conversely, RBP2 knockdown markedly increased H3K4me3 levels at all three motifs (all *P* < 0.0001) (**Figures 2L–N**). Notably, the site of strongest RBP2 binding (motif 3) did not coincide with the site of most significant H3K4me3 change (motif 2). This indicates that RBP2’s demethylase activity is not simply proportional to its binding intensity, but is modulated by the local chromatin environment. Specifically, it is the boundary region of the H3K4me3 peak, where steady-state methylation levels are lower and more susceptible to demethylation. Furthermore, this H3K4me3 reduction occurred concomitantly with increased BECN1 expression, an apparent paradox given that H3K4me3 is generally associated with active transcription. Together, these data indicate that while RBP2 directly binds to the BECN1 promoter and possesses demethylase activity at this locus, the transcriptional activation of BECN1 by RBP2 is unlikely to be mediated by H3K4me3-dependent mechanisms.

**Figure 3:**
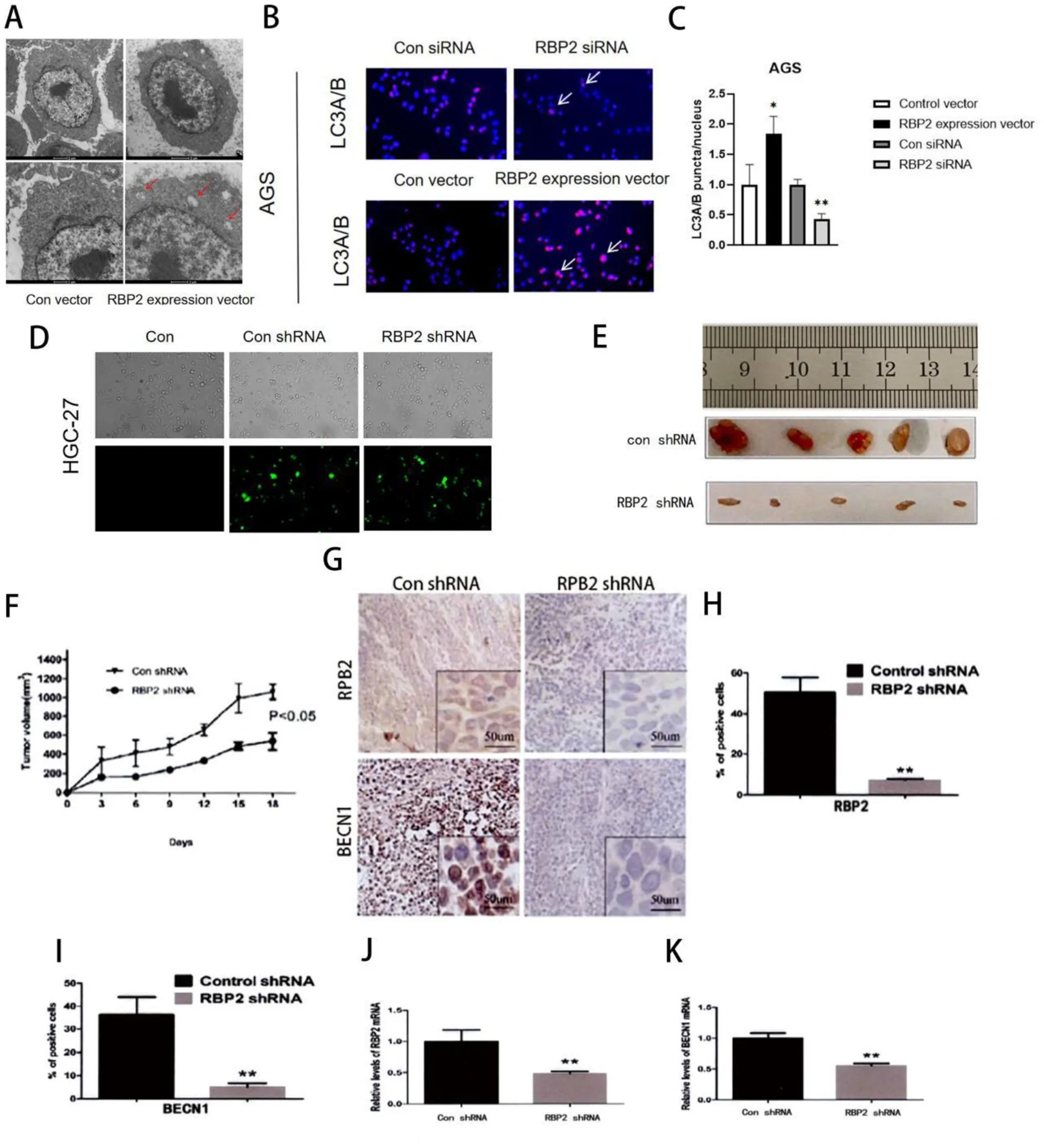
RBP2 induces autophagy and promoted tumor growth *in vivo*. **(A)** Transmission electron microscopy (TEM) images showing autophagosome formation (red arrows) in GC cells. Scale bar, 2 μm, 1 μm. **(B, C)** Representative immunofluorescence images (20×, B) and LC3A/B puncta formation in RBP2-overexpressed or knocked-down cells **(C)** of quantitative analysis. **(D)** Schematic illustration of the xenograft tumor model using stable RBP2-knockdown HGC-27 cells (20×). **(E, F)** Tumor morphology and growth curve of xenograft tumors. **(G-K)** IHC staining and qRT-PCR validation of RBP2 and BECN1 expression in xenograft tissues. Scale bar, 50 μm. Data are mean ± SD. *P < 0.05, **P < 0.01.

Given that BECN1 is the core initiator of autophagy, we assessed autophagic flux. TEM revealed that RBP2 overexpression (**Supplementary Figures 2A, B**) increased the number of double-membrane autophagosomes (**Figure 3A**). Consistently, immunofluorescence staining for LC3A/B showed enhanced puncta formation upon RBP2 overexpression, which was abolished by RBP2 knockdown (**Supplementary Figures 2C, D**, **Figures 3B, C**). *In vivo*, stable RBP2 knockdown in HGC-27 cells suppressed xenograft tumor growth, reducing both tumor volume and weight (**Figures 3D–F**). IHC and qRT-PCR analysis of harvested tumors confirmed that RBP2 depletion downregulated BECN1 expression (**Figures 3G–K**). Thus, RBP2 drives autophagy in GC through transcriptional regulation of BECN1.

### 3.2 The RBP2-BECN1 autophagy axis contributes to VEGFA secretion in GC cells

**Figure 4:**
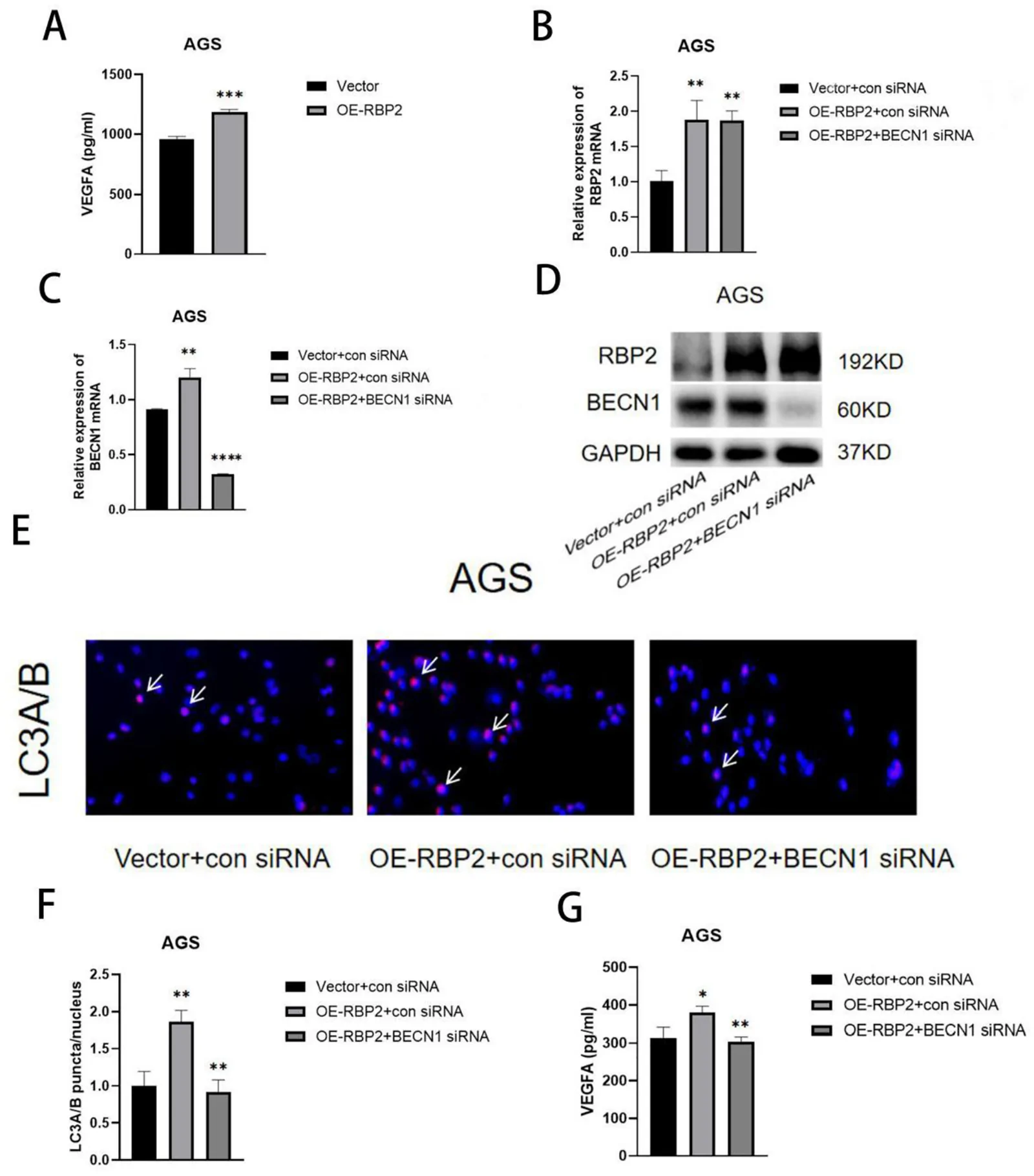
The RBP2*-*BECN1-autophagy axis drives VEGFA secretion. **(A)** ELISA quantification of VEGFA secretion in the supernatant of RBP2-overexpressed GC cells. **(B-D, G)** Rescue assays showing that BECN1 knockdown reversed RBP2-induced VEGFA secretion. **(E, F)** Representative immunofluorescence images (20×) and quantification confirming that BECN1 depletion abolished RBP2-induced LC3 puncta formation. Data are mean ± SD. *P< 0.05, **P < 0.01, ***P < 0.001, ****P < 0.0001.

We previously reported that RBP2 directly binds to the *VEGFA* promoter and transcriptionally activates its expression in GC cells (28). However, whether RBP2 regulates VEGFA through additional, indirect mechanisms remained unknown. Given our finding that RBP2 also transcriptionally upregulates the core autophagy driver BECN1, and accumulating evidence that autophagic flux can promote VEGFA secretion under certain conditions, we hypothesized that RBP2 might drive VEGFA production through a two-pronged mechanism: direct transcriptional activation and autophagy-mediated secretion. To test whether the BECN1-autophagy axis contributes to RBP2-induced VEGFA secretion, we confirmed that ectopic RBP2 expression in GC cells significantly induced canonical autophagy activation, evidenced by a significant increase in LC3A/B ratio and dense cytosolic LC3 puncta formation (**Figures 3B, C**), concurrent with a distinct elevation of VEGFA secretion into culture supernatants quantified by ELISA (**Figure 4A**). To establish causality, we performed rescue assays by knocking down BECN1 in the context of RBP2 overexpression (**Figures 4B–D**), which abrogated RBP2-induced autophagy flux with loss of LC3 puncta (**Figures 4E, F**) and simultaneously reversed the RBP2-driven VEGFA secretion level increase (**Figure 4G**). Together, these data demonstrate that RBP2 promotes VEGFA secretion at least in part through the downstream BECN1-autophagy cascade, which functions as a mandatory intermediate for the autophagy-dependent component of VEGFA regulation. This mechanism operates in parallel with, and independently of, the direct transcriptional activation of VEGFA by RBP2 that we reported previously (28), formalizing the RBP2→BECN1(autophagy)→VEGFA (angiogenesis) signaling arm of the broader regulatory circuit.

### 3.3 VEGFA feeds back to upregulate RBP2 via the JNK/p53/miR-212 axis, forming a potential positive regulatory circuit

**Figure 5:**
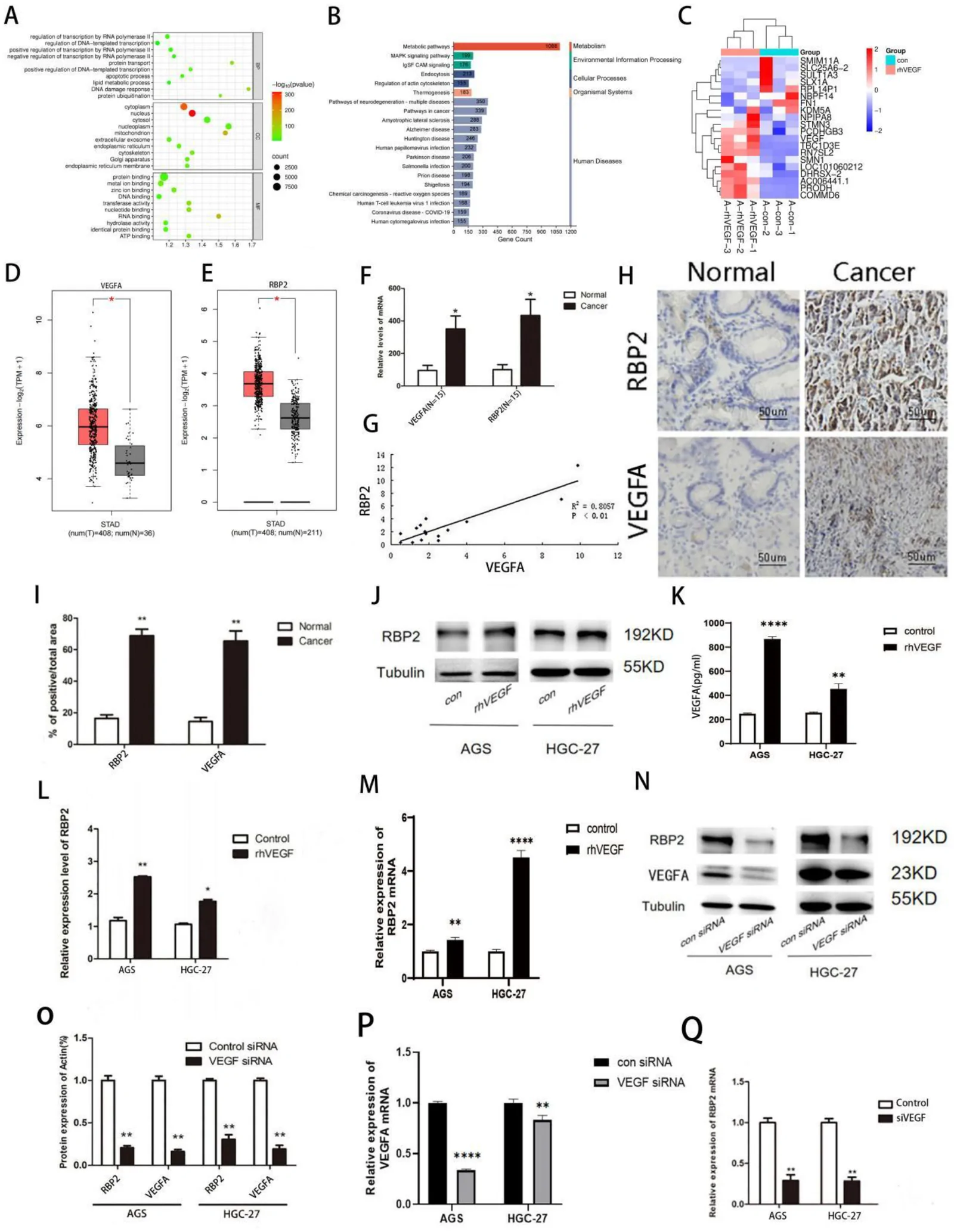
VEGFA positively correlates with RBP2 and upregulated its expression. **(A, B)** Transcriptome profiling of rhVEGF-treated GC cells, showing enrichment of RBP2-related oncogenic pathways via KEGG/GO analysis. **(C)** Heatmap analysis of VEGFA-regulated potential target genes. **(D, E)** Analysis of VEGFA and RBP2 expression in GC tissues versus normal tissues using the UALCAN-TCGA and GTEx data via GEPIA2. **(F-I)** IHC analysis of VEGFA and RBP2 expression in 15 paired GC specimens, demonstrating a strong positive correlation (R = 0.8057, P< 0.01). Scale bar, 50 μm. **(J-Q)** *In vitro* validation showing that exogenous rhVEGF treatment upregulated RBP2 mRNA and protein levels, whereas endogenous VEGFA knockdown reduced​ them. Data are mean ± SD. *P < 0.05, **P < 0.01, ****P < 0.0001.

Having established that RBP2 drives VEGFA secretion via BECN1-dependent autophagy, we next asked whether VEGFA might reciprocally regulate RBP2 expression, forming a feedback loop. Such bidirectional regulation would have important implications for tumor progression and therapeutic resistance. To first test whether VEGFA can influence RBP2 expression at the transcriptome level, we performed RNA-seq on rhVEGF-treated AGS cells. Transcriptome profiling suggested that VEGFA modulates RBP2 expression via indirect signaling cascades (**Figures 5A–C**, **Supplementary Figure 3A**). Analysis of the UALCAN-TCGA and GTEx datasets revealed that both VEGFA and RBP2 are significantly upregulated in GC tissues compared to adjacent normal tissues (**Figures 5D, E**). Supporting this, 15 paired clinical specimens exhibited a strong positive correlation between VEGFA and RBP2 expression (*R* = 0.8057, *P* < 0.01) (**Figures 5F–I**). Exogenous rhVEGF treatment upregulated RBP2 mRNA and protein levels, whereas endogenous VEGFA knockdown reduced them (**Figures 5J–Q**).

**Figure 6:**
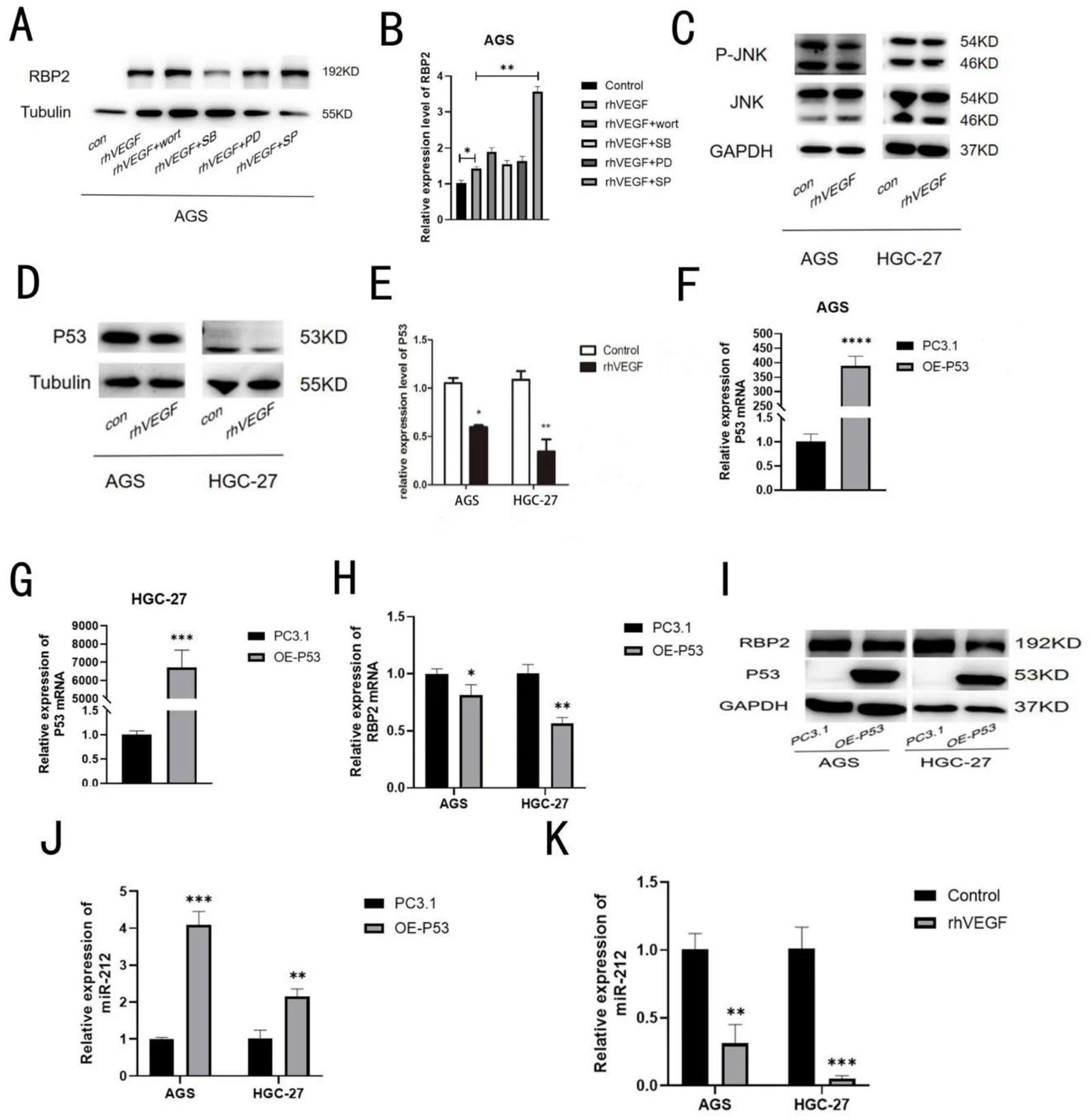
VEGFA upregulates RBP2 via the JNK/p53/miR-212 signaling axis. **(A, B)** Screening of VEGFA downstream pathways using specific inhibitors. Only the JNK inhibitor SP600125 enhanced rhVEGF-induced RBP2 upregulation. **(C-E)** Western blot analysis showing rhVEGF suppressed JNK phosphorylation and downregulated p53 expression. **(F-K)** Mechanism dissection showing p53 inhibited RBP2 expression and positively regulated miR-212 expression, while rhVEGF treatment reduced miR-212 levels. Data are mean ± SD. *P< 0.05, **P< 0.01, ***P< 0.001, ****P< 0.0001.

Having confirmed that VEGFA upregulates RBP2, we next sought to identify the downstream signaling pathway responsible. VEGFA is known to activate multiple canonical pathways including PI3K/AKT, MEK/ERK, p38 MAPK, and JNK (29, 30). To determine which pathway mediates VEGFA-induced RBP2 upregulation, we employed a pharmacological inhibitor screening approach. Surprisingly, only the JNK inhibitor SP600125 enhanced rhVEGF-induced RBP2 upregulation, unlike inhibitors of PI3K/AKT, ERK, or p38 MAPK, which had no significant effect (**Figures 6A, B**). This result was unexpected and suggested that JNK acts as a negative regulator of RBP2 in this context, and that VEGFA functions to inhibit JNK signaling. Consistent with this model, rhVEGF treatment suppressed JNK phosphorylation (**Figure 6C**). To understand how JNK negatively regulates RBP2, we turned to the well-established connection between JNK and the tumor suppressor p53. JNK is known to phosphorylate p53 at multiple sites (including Ser15 and Ser33), which stabilizes p53 protein by disrupting its interaction with MDM2 and promoting its nuclear accumulation (31). We therefore hypothesized that VEGFA-mediated JNK inhibition would lead to reduced p53 stability and lower p53 protein levels. In fact, this suppression led to a marked downregulation of p53 protein levels (**Figures 6D, E**). We next asked how p53 downregulation leads to RBP2 upregulation. Our prior work identified miR-212 as a direct repressor of RBP2 that binds to the 3′-UTR of RBP2 mRNA (32). Importantly, p53 has been shown to transcriptionally activate miR-212 expression through p53 response elements in the miR-212 promoter region. We therefore tested the following cascade: VEGFA → JNK inhibition → p53 destabilization → reduced miR-212 transcription → de-repression of RBP2. We found that p53 positively regulates miR-212 expression, and rhVEGF treatment significantly reduced miR-212 levels in GC cells (**Figures 6F–K**). Together, these data delineate a cascade whereby VEGFA inhibits JNK/p53 to relieve miR-212-mediated repression of RBP2.

**Figure 7:**
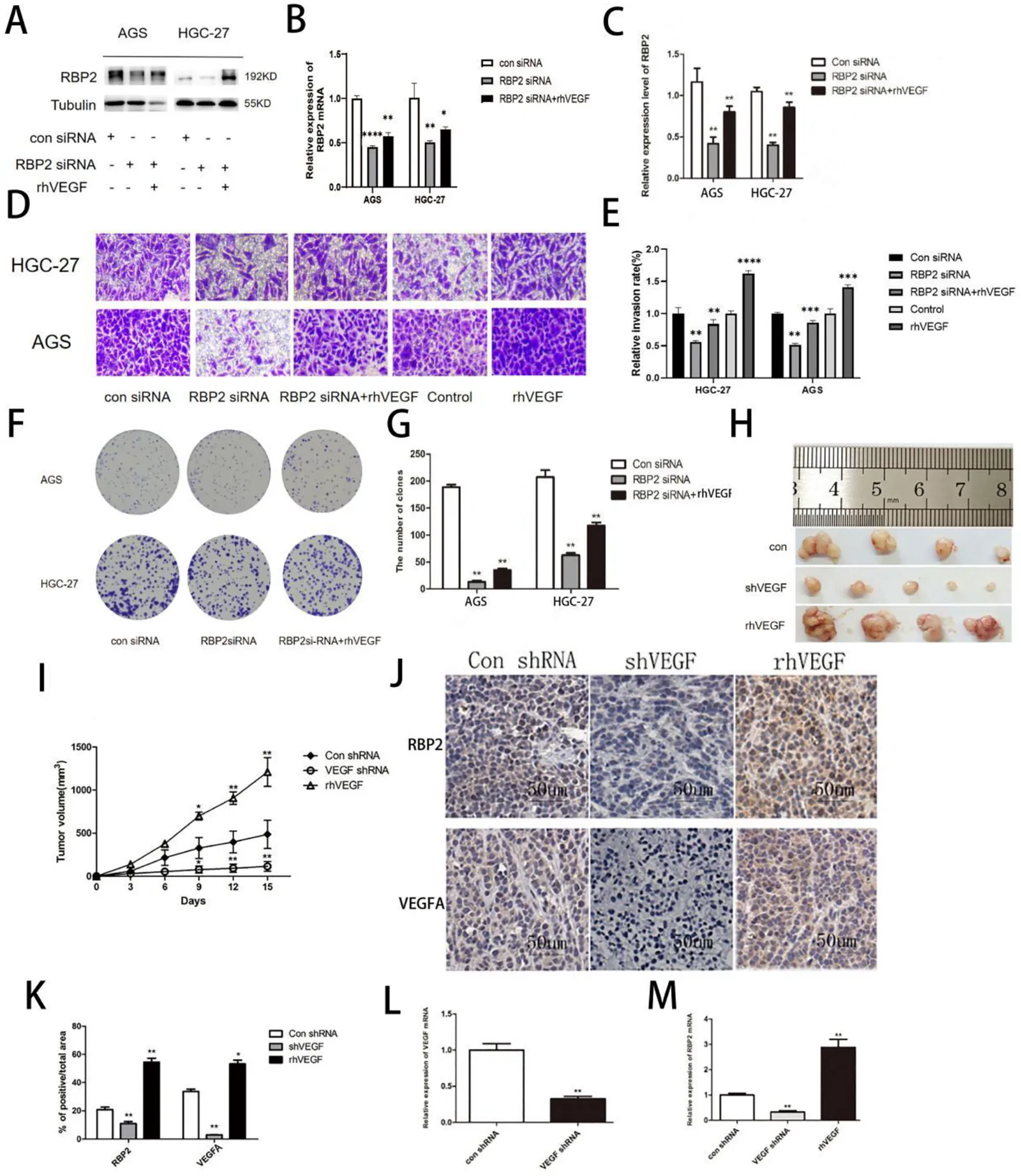
The VEGFA-RBP2 feedback loop promotes GC malignant progression. **(A-C)** RBP2 expression in rescue experiments. **(D, E)** Transwell invasion assays showing rhVEGF pretreatment rescued the invasive capacity of RBP2-knockdown cells. **(F, G)** Colony formation assays quantifying the rescue effect on cell proliferation. **(H, I)** Xenograft tumor growth curves and excised tumors from mice treated with VEGFA knockdown or rhVEGF supplementation. **(J-M)** IHC staining and qRT-PCR detection of VEGFA and RBP2 levels in xenograft tissues. Scale bar, 50 μm. Data are mean ± SD. *^ns^P*>0.05, *P < 0.05, **P < 0.01, ***P < 0.001, ****P < 0.0001.

Functional rescue assays demonstrated that rhVEGF pretreatment restored the proliferative and invasive capacity of RBP2-knockdown cells (**Figures 7A–G**). *In vivo*, VEGFA knockdown suppressed tumor growth and RBP2 expression, while rhVEGF supplementation promoted both (**Figures 7H–M**). Taken together, these findings support a self-amplifying regulatory circuit: RBP2↑ → BECN1↑ → autophagy↑ → VEGFA↑ → JNK/p53/miR-212 inhibition → RBP2↑↑, which sustains GC progression.

## 4 Discussion

Accumulating evidence positions histone demodifiers not as passive chromatin erasers but as active rheostats that translate microenvironmental signals into transcriptional outputs (33, 34). In this study, our data support a self-amplifying regulatory model, RBP2 → BECN1 (autophagy) → VEGFA (angiogenesis) → JNK/p53/miR-212 → RBP2, that drives GC progression. Rather than acting solely through canonical H3K4me3 demethylation-dependent repression, RBP2 functions in our model as a transcriptional regulator of BECN1, establishing a feed-forward circuit that couples epigenetic reprogramming to autophagic flux and VEGFA-dependent paracrine signaling.

RBP2 exerts context-dependent transcriptional outputs that extend beyond its canonical role as a repressor. While RBP2 is traditionally characterized as a transcriptional suppressor via H3K4me3 demethylation at target promoters (35–37), our data demonstrate that it transcriptionally regulates BECN1 in GC, supported by both dual-luciferase reporter assays and ChIP-qPCR evidence of physical promoter occupancy. Such context-dependent functional switching is consistent with emerging evidence that KDM5 family members can engage transcriptional activation machinery through context-specific cofactors or pioneer factor partnerships (38, 39), placing BECN1 within this “activator” compartment. To our knowledge, this is the first demonstration that RBP2 transcriptionally regulates an autophagy-initiating gene in GC.

A central question arising from our data is how RBP2, a well-characterized H3K4me2/3 demethylase, can transcriptionally activate BECN1. Our ChIP-qPCR data reveal an uncoupling between RBP2 occupancy and its demethylase activity at the BECN1 promoter. RBP2 binding is strongest at motif 3 (−205/−200), the most proximal site located within the H3K4me3 peak, yet the most significant H3K4me3 reduction upon RBP2 overexpression occurs at motif 2 (−261/−256), which lies at the upstream boundary of the H3K4me3 peak. This indicates that RBP2’s promoter recruitment and its catalytic demethylase activity are not strictly proportional, but are modulated by the local chromatin environment. Furthermore, the overall H3K4me3 reduction occurs concomitantly with increased BECN1 expression, an apparent paradox given that H3K4me3 is universally associated with active transcription, suggesting that RBP2 activates BECN1 largely through a demethylase-independent mechanism.

Several non-mutually exclusive models may explain this uncoupling. RBP2 could function primarily as a chromatin reader or scaffold at the proximal promoter within the H3K4me3 peak (motif 3), recruiting co-activator complexes via its ARID/PHD domains to drive transcription independently of demethylation, while its canonical demethylase activity predominates at the peak boundary (motif 2) where local methyltransferase activity is lower and steady-state H3K4me3 levels are more susceptible to net reduction. Alternatively, this boundary effect may reflect substrate homeostasis. The core H3K4me3 peak is actively maintained by methyltransferases such as MLL/SET complexes, making it more resistant to demethylation than peripheral boundary regions. Regardless of the precise mechanism, these data suggest unanticipated functional complexity at a single locus and challenge the simple view of KDM5 proteins as purely repressive epigenetic regulators, further supporting the emerging concept of histone demethylases as context-dependent transcriptional rheostats rather than binary on/off switches (33, 34).

Beyond the mechanistic details of RBP2-mediated BECN1 regulation, this self-amplifying circuit also reframes how autophagy and angiogenesis are coordinated in GC. While VEGFA typically signals through receptor tyrosine kinases to activate canonical downstream effectors such as PI3K/AKT, MEK/ERK, and p38 MAPK in various pathophysiological contexts (29, 30), our pharmacological inhibitor screen revealed a distinct requirement for JNK signaling in mediating VEGFA-induced RBP2 upregulation. Prior work links autophagic flux to VEGFA secretion under metabolic stress (40–43). However, most models treat this as a one-way, stress-responsive output. We show that RBP2-induced autophagy not only raises VEGFA production but that secreted VEGFA feeds back to sustain its own epigenetic driver (RBP2), generating a self-reinforcing architecture. Notably, this circuit operates through JNK inactivation, p53 loss, and miR-212 downregulation, bypassing the canonical HIF-1α-mediated hypoxia axis in our normoxic culture system. Whether this loop intersects with or substitutes for hypoxic drivers *in situ* remains to be delineated.

It is important to distinguish the present findings from our earlier work demonstrating that RBP2 directly transactivates VEGFA to promote angiogenesis in GC (28). The current study extends this prior observation in three significant ways. First, we identify a parallel, autophagy-dependent pathway through which RBP2 drives VEGFA production, specifically via transcriptional upregulation of BECN1 and subsequent activation of autophagic flux. This mechanism is distinct from the direct promoter binding to VEGFA reported previously. Second, we demonstrate that these two pathways (direct transcriptional activation and autophagy-mediated secretion) are not mutually exclusive, rather they may function cooperatively to amplify VEGFA output. Third, and most importantly, we uncover a reciprocal feedback loop in which VEGFA signals back to upregulate RBP2 via the JNK/p53/miR-212 axis, creating a self-sustaining circuit that was entirely unrecognized in our earlier work. Thus, the present study transforms our understanding from a one-way linear relationship (RBP2 → VEGFA) into a bidirectional, self-amplifying network with profound implications for tumor resilience and therapeutic resistance.

From a translational angle, the JNK/p53/miR-212 limb warrants further preclinical investigation as a potential therapeutic target. Anti-VEGFA agents, such as bevacizumab, have yielded modest and inconsistent benefits in GC (AVAGAST/GLOW trials) (44, 45) and adaptive survival programs are a suspected contributor. Our data suggest a hypothesis that requires rigorous validation that VEGFA blockade could trigger compensatory RBP2 stabilization via the same JNK/p53/miR-212 axis we describe, and combined disruption, for example, VEGFA signaling plus autophagy inhibition (hydroxychloroquine or later-generation lysosomal blockers) or JNK pathway restoration, might circumvent such compensation. However, these translational implications remain speculative at this stage and should be interpreted with caution, as they are based on *in vitro* and subcutaneous xenograft data only. Critically, because JNK exerts opposing effects depending on cellular context, promoting inflammation and surveillance in some stromal compartments while supporting survival in others (31) any such strategy would require careful window definition.

Several limitations of our current study warrant consideration. First, although we validated the loop in GC cell lines and subcutaneous xenografts, the spatial architecture of this axis within the native tumor microenvironment, such as whether it is enriched in hypoxic necrotic cores, invasive fronts, or specific Lauren subtypes, awaits validation using patient-derived organoid (PDO) and orthotopic models. Second, while our pharmacological screening pinned the VEGFA → RBP2 relay on JNK, the upstream receptor (VEGFR1 *vs*. VEGFR2) and potential co-receptors mediating this suppression need clarification. Third, high-resolution mapping approaches such as CUT&Tag or ChIP-exo will be required to define the precise binding nucleotides and whether RBP2 binds directly via its ARID/PHD domains or indirectly through co-regulator complexes. Fourth, catalytically dead RBP2 mutants (e.g., H483A) and chromosome conformation capture assays (3C or Hi-C) will be needed to formally test the demethylase-independent activation model and the role of long-range chromatin looping. Finally, selective KDM5 inhibitors are emerging (22, 46, 47), but their impact on autophagy-angiogenesis coupling, and their window of safety given RBP2’s roles in normal tissue homeostasis, requires systematic evaluation before translational claims can be made.

**Figure 8:**
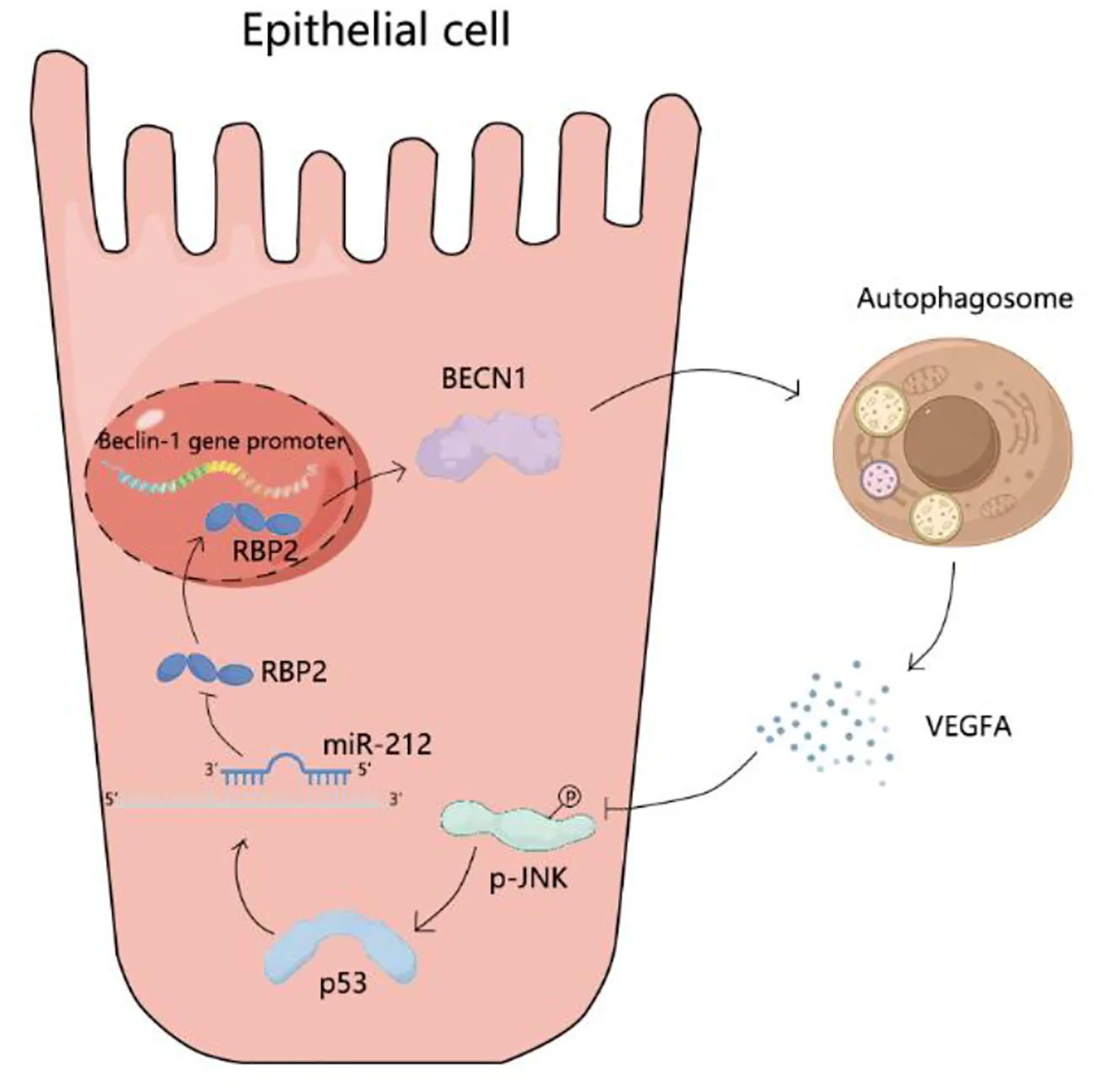
Proposed model of the RBP2-BECN1-VEGFA regulatory circuit. RBP2 transcriptionally regulates *BECN1* to trigger cytoprotective autophagy, which further facilitates VEGFA secretion. Secreted VEGFA in turn upregulates RBP2 expression via repressing the JNK/p53/miR-212 signaling axis, thereby forming a self-amplifying regulatory circuit that persistently promotes the survival, proliferation and invasion of gastric carcinoma.

In conclusion, our findings support the existence of a self-sustaining epigenetic-autophagic-angiogenic circuit that drives GC progression. By wiring transcriptional activation (RBP2→BECN1), cytoprotective autophagy, and growth factor feedback into one loop, GC cells appear to build a resilient, self-fueling network. Targeting this circuitry through rational combinations, rather than empirical stacking, represents a biologically grounded direction that merits further preclinical investigation (**Figure 8**).

## Data Availability

The data discussed in this publication have been deposited in NCBI’s Gene Expression Omnibus (Edgar et al., 2002) and are accessible through GEO Series accession number GSE334190 (https://www.ncbi.nlm.nih.gov/geo/query/acc.cgi?acc=GSE334190).
