# Supplementary material for: The RBP2–BECN1–VEGFA axis orchestrates a self-amplifying circuit to drive malignant progression in gastric carcinoma
Source: Front Oncol. 2026 Sep 7;16:1929764. doi: 10.3389/fonc.2026.1929764 (PMC13590304; doi:10.3389/fonc.2026.1929764)

Figure 1K

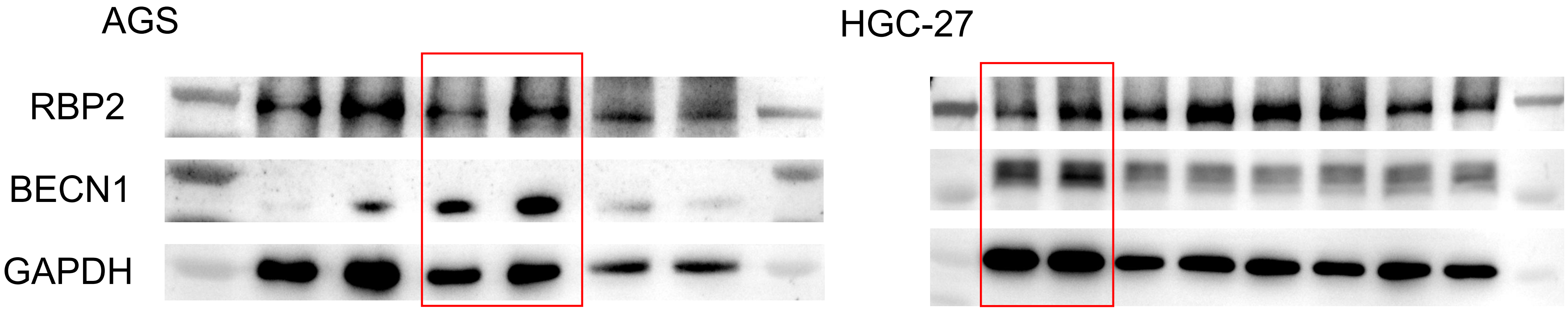

Figure 1O

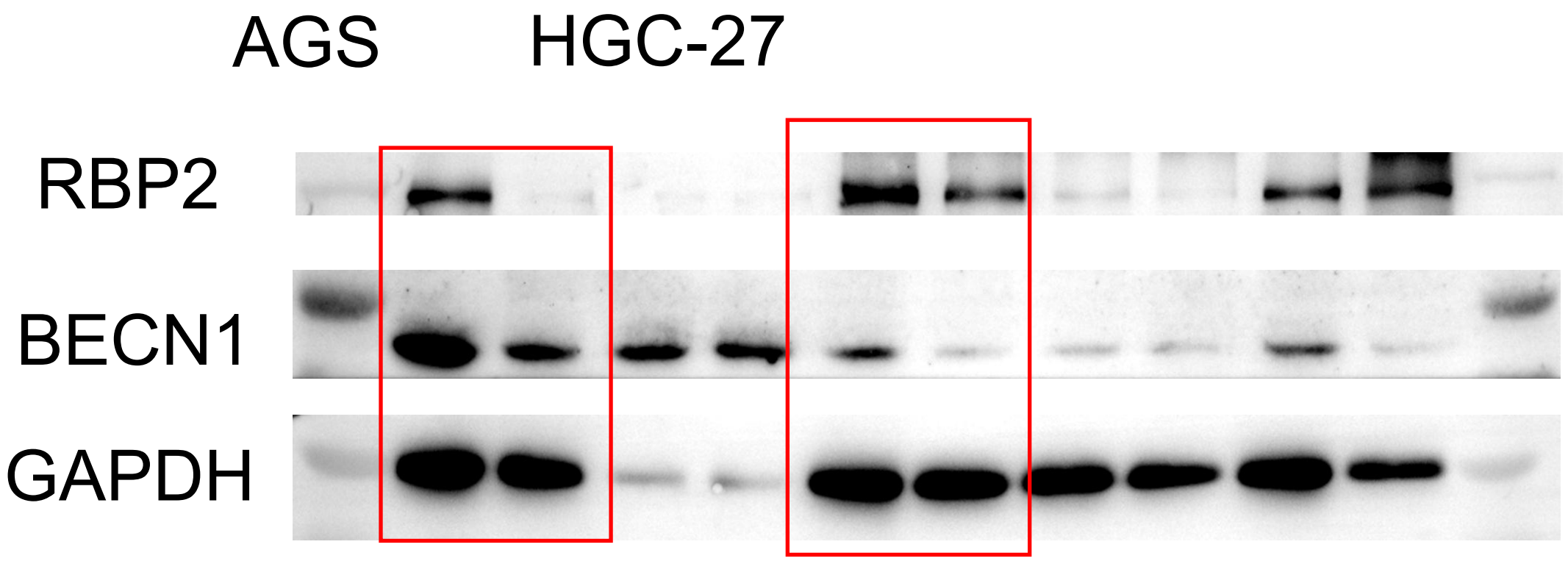

Figure 3D

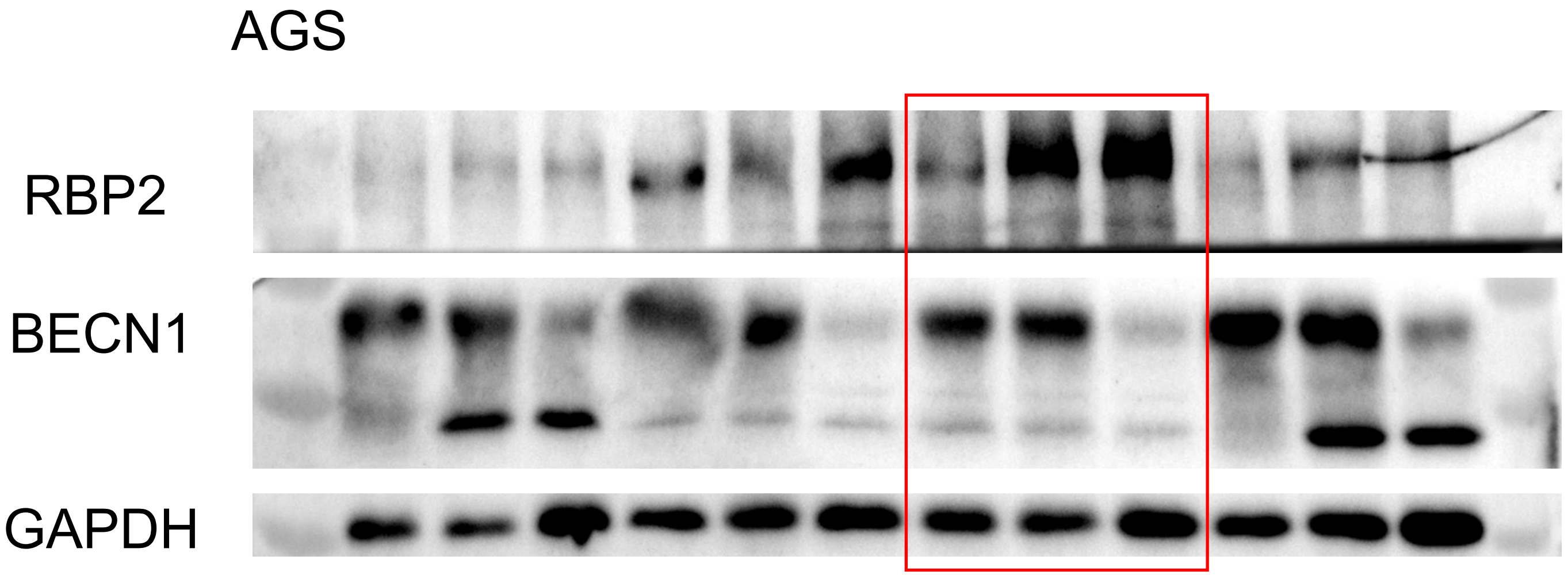

Figuer 4J

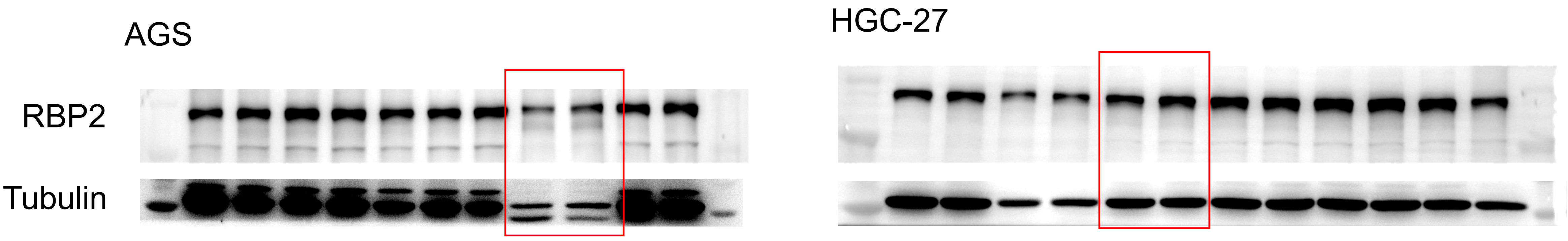

Figuer 4N

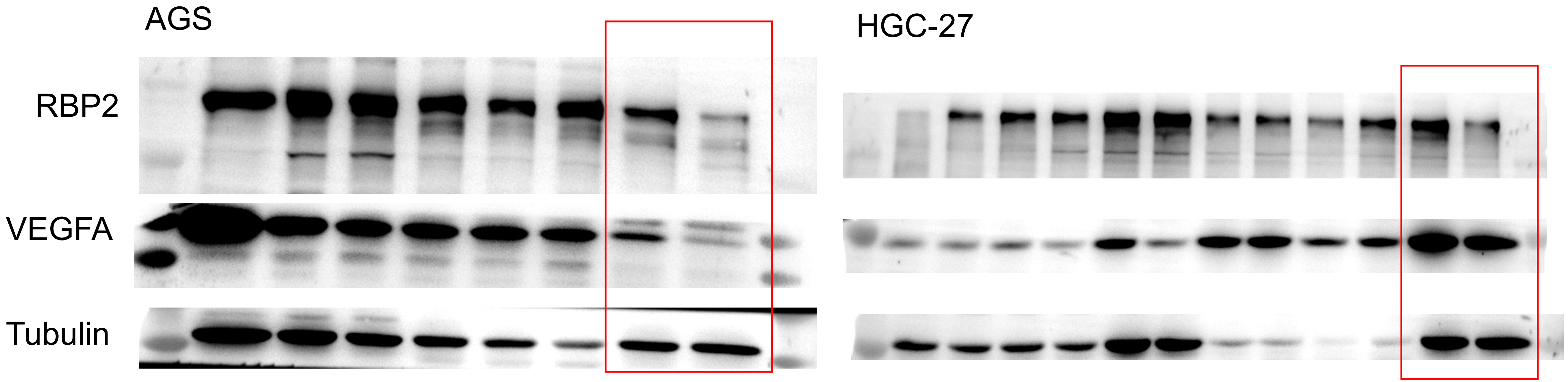

Figure 5A

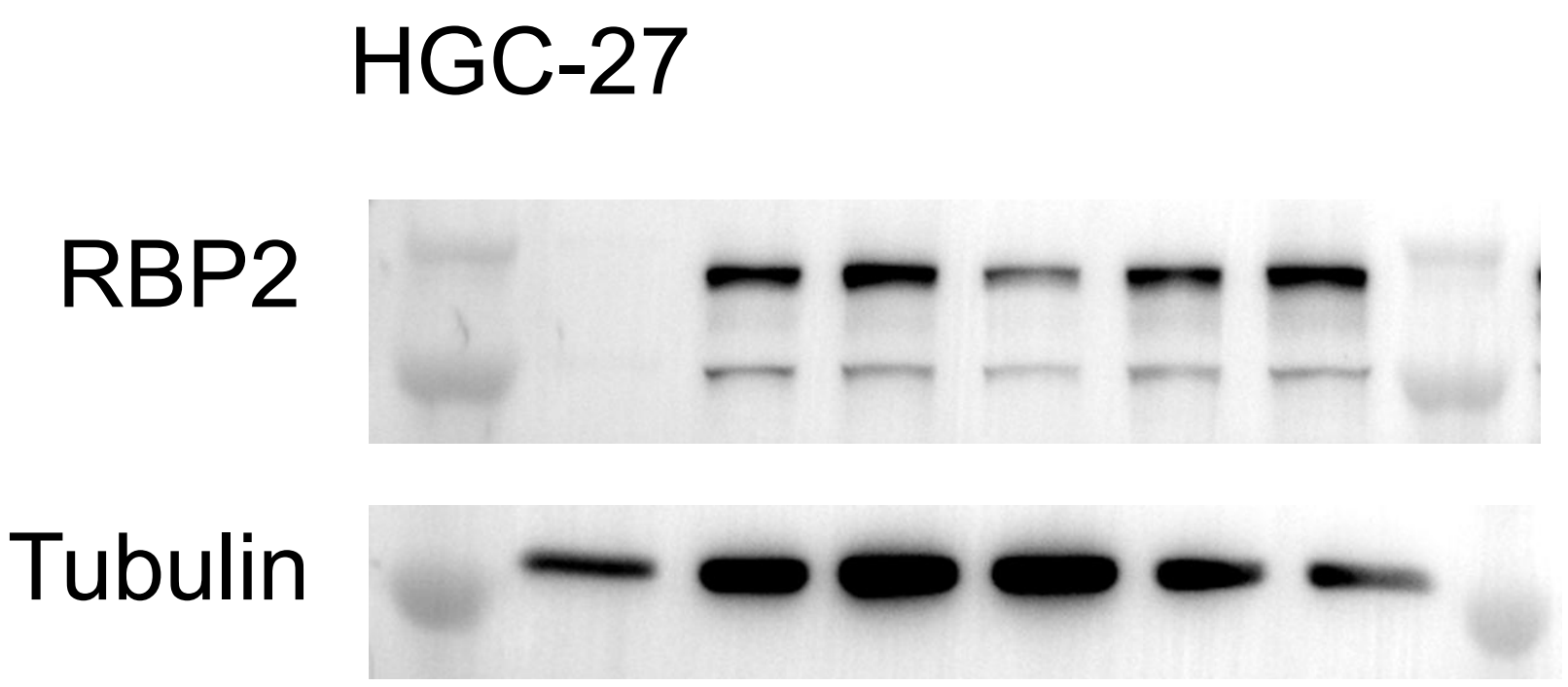

Figure 5C

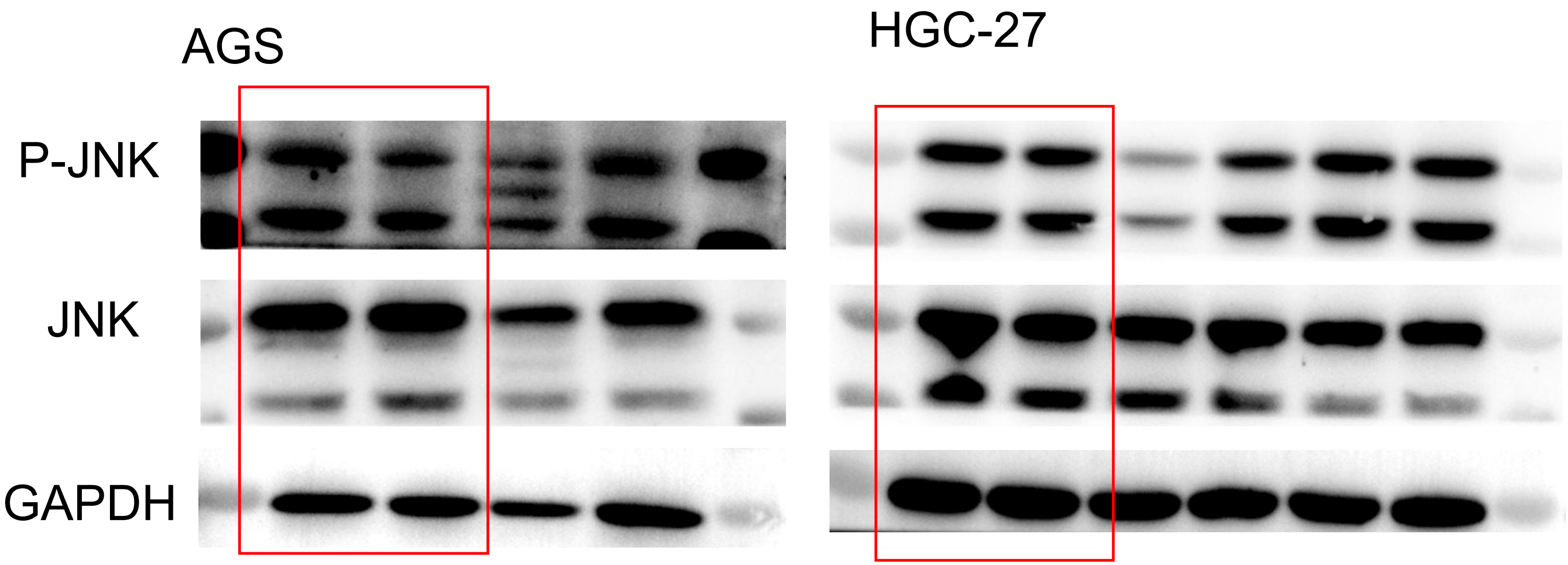

Figure 5D

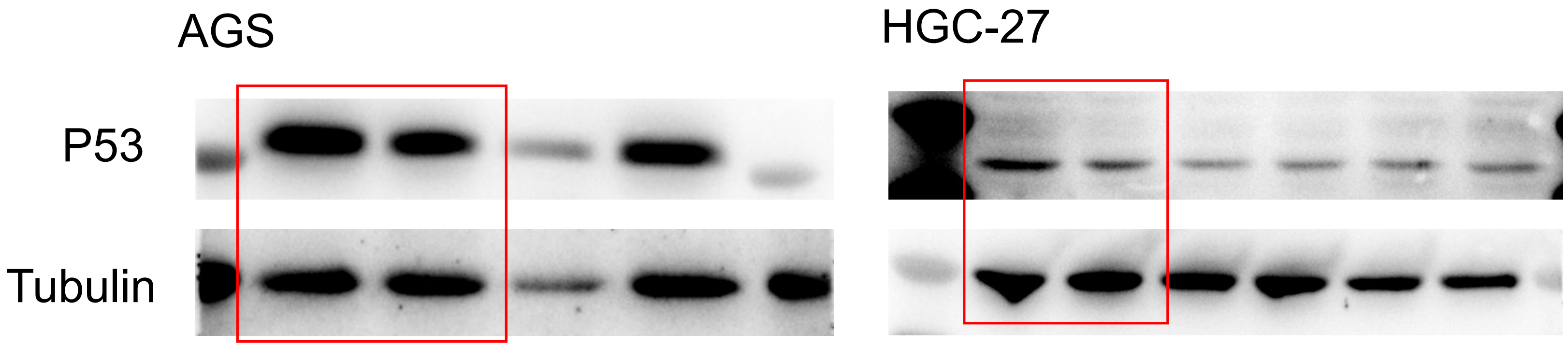

Figure 5I

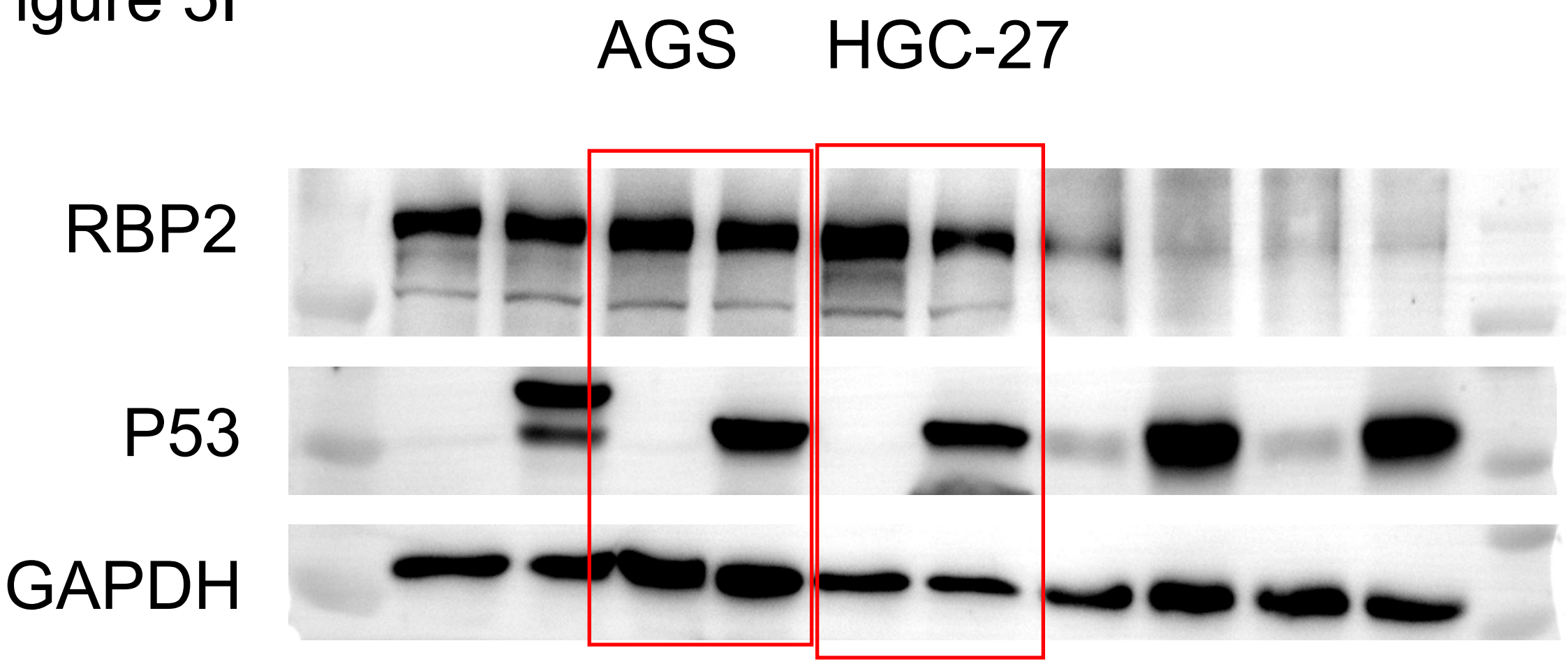

Figure 6A

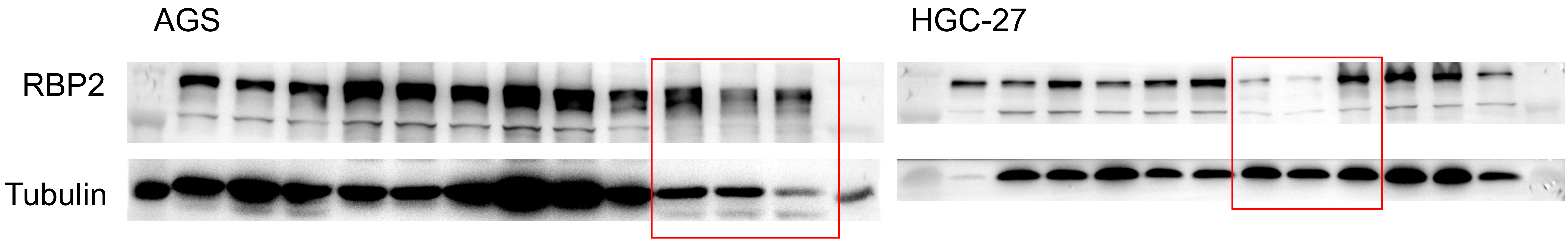

Figure S1A

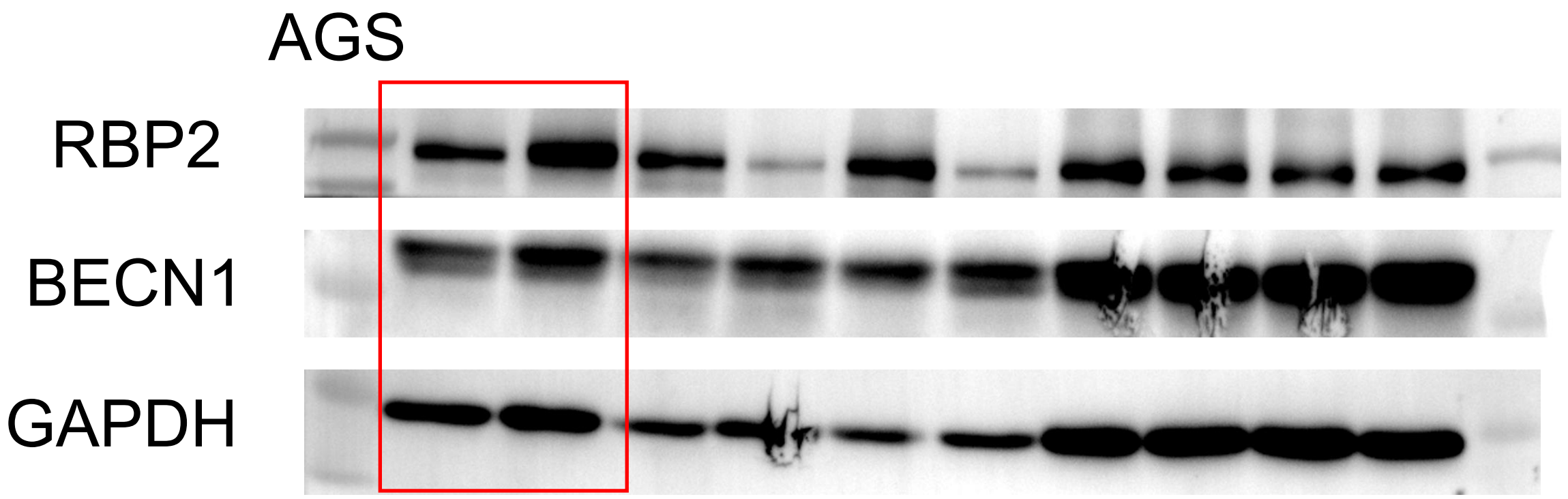

Figure S1C

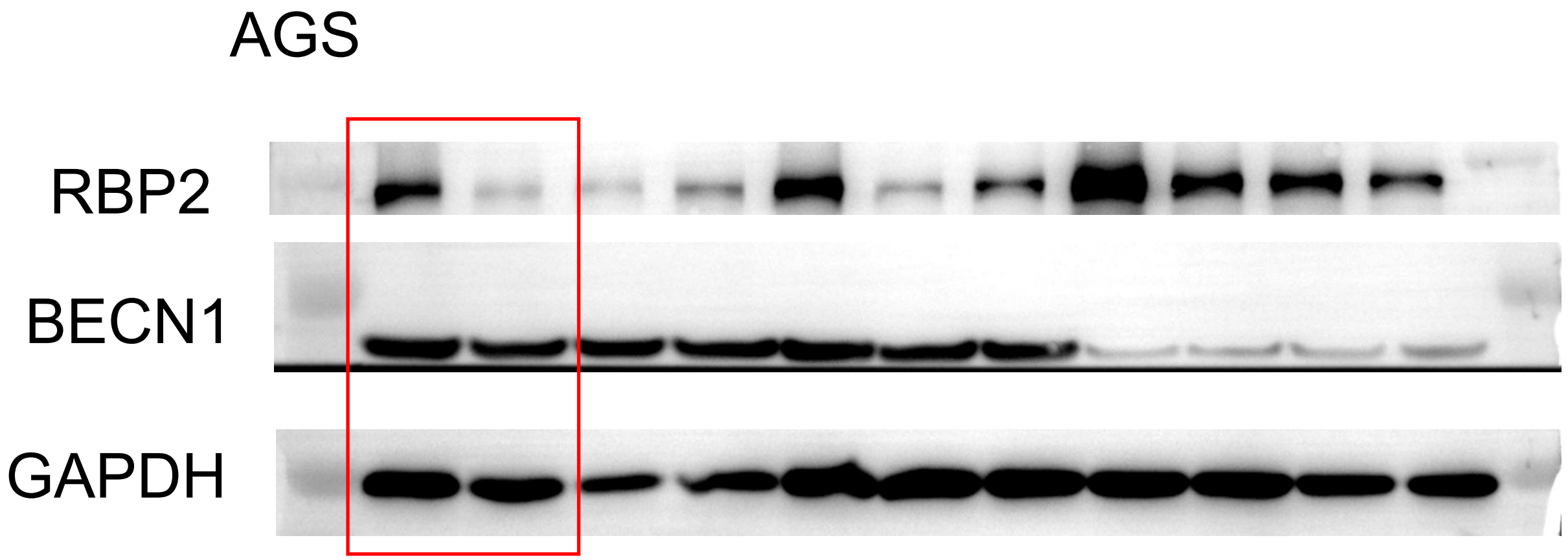

Supplement: Supplementary file 1 [file Supplementaryfile2.pdf]
