# Supplementary material for: The RBP2–BECN1–VEGFA axis orchestrates a self-amplifying circuit to drive malignant progression in gastric carcinoma
Source: Front Oncol. 2026 Sep 7;16:1929764. doi: 10.3389/fonc.2026.1929764 (PMC13590304; doi:10.3389/fonc.2026.1929764)

Figure 2C

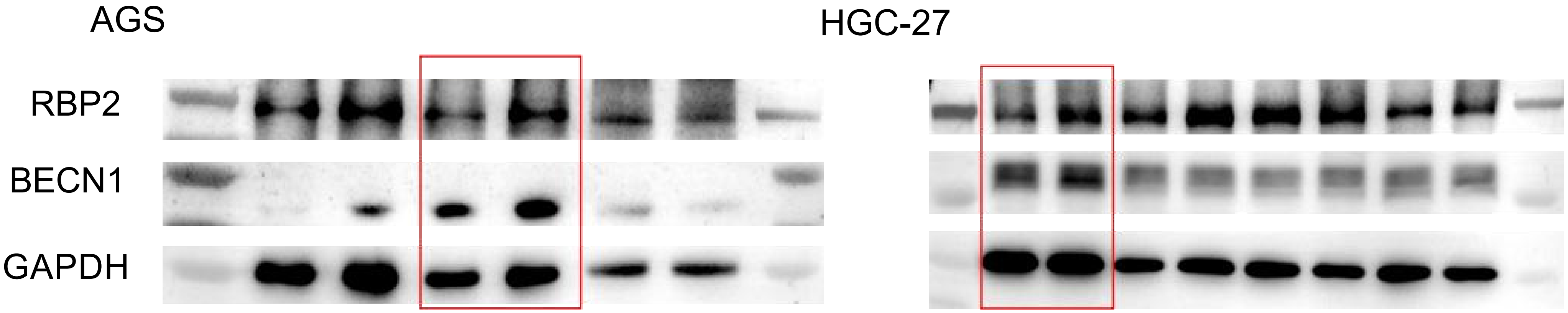

Figure 2G

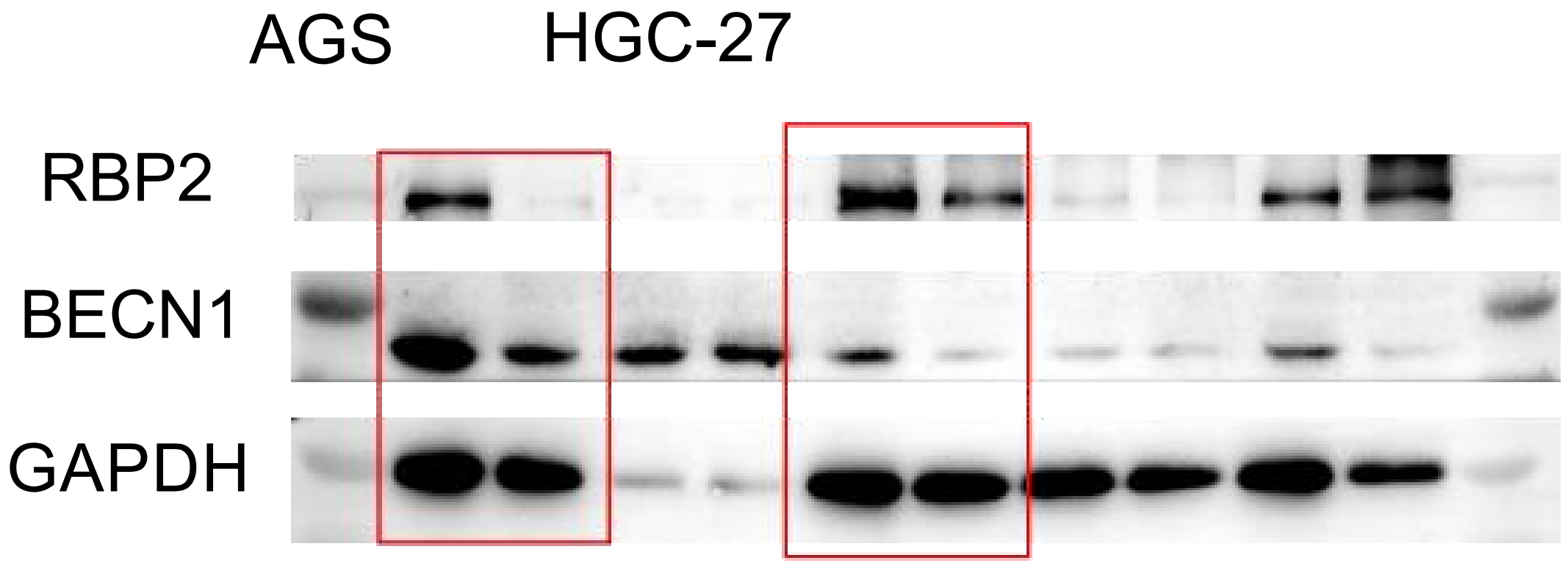

Figure 4D

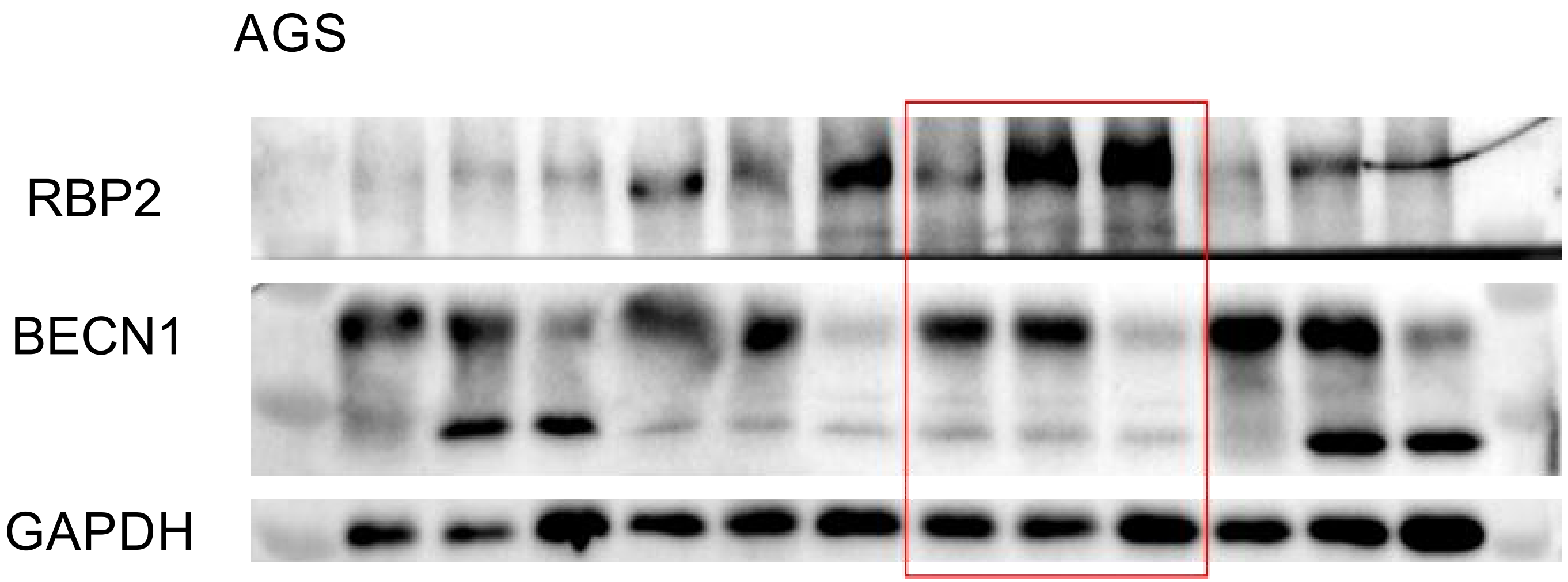

Figuer 5J

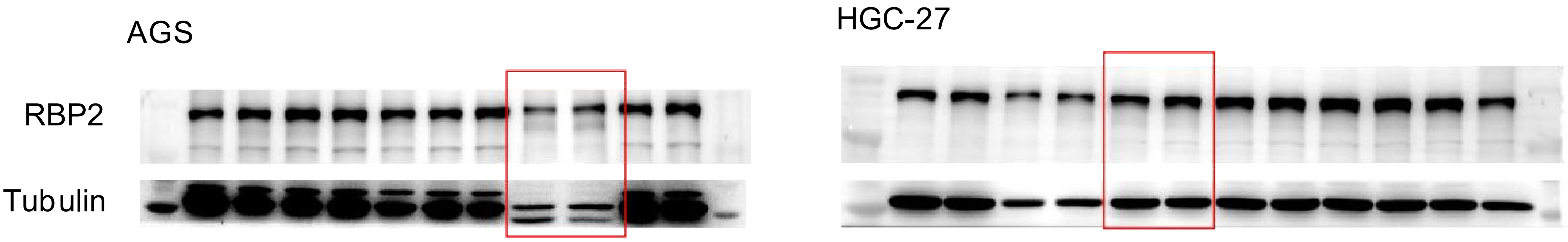

Figuer 5N

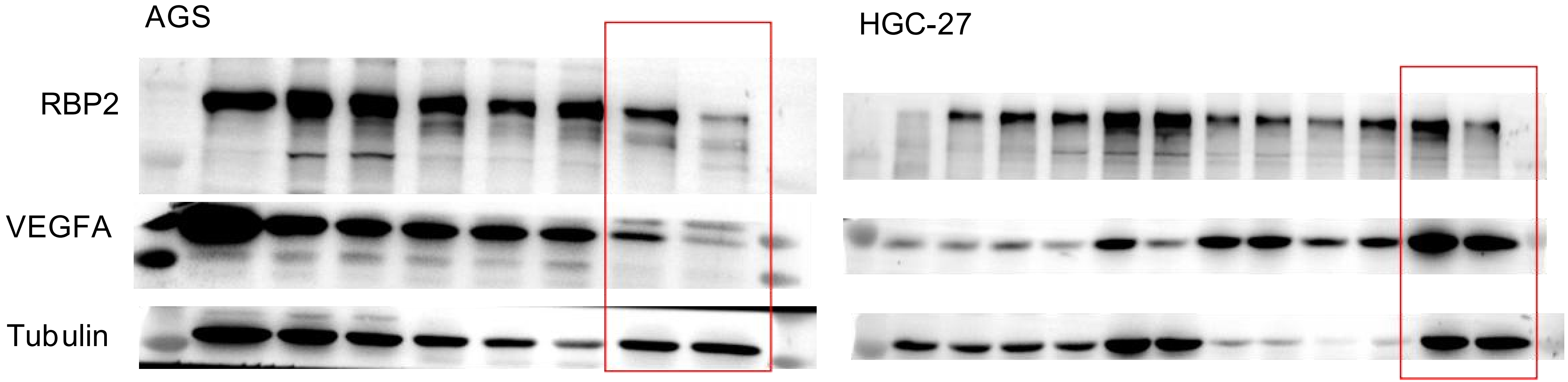

Figure 6A

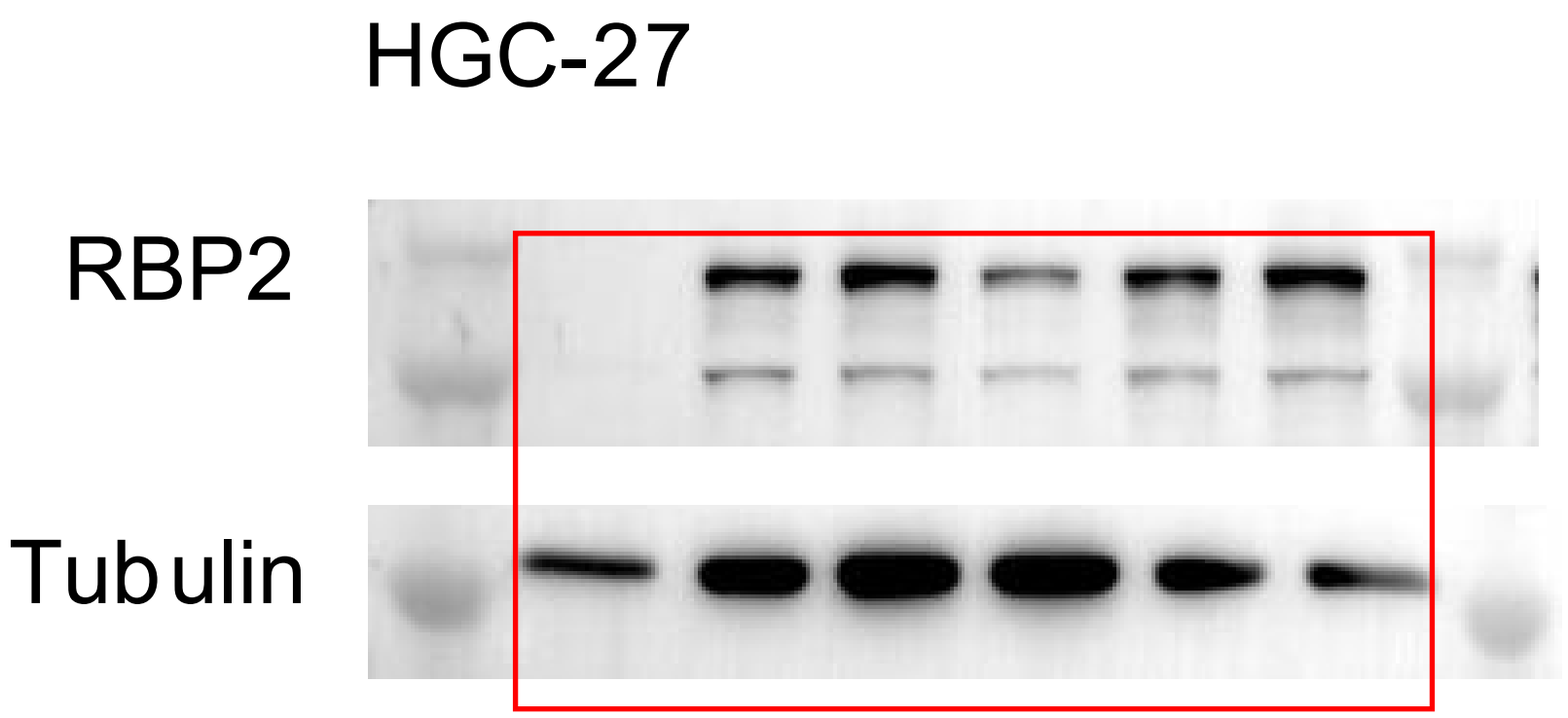

Figure 6C

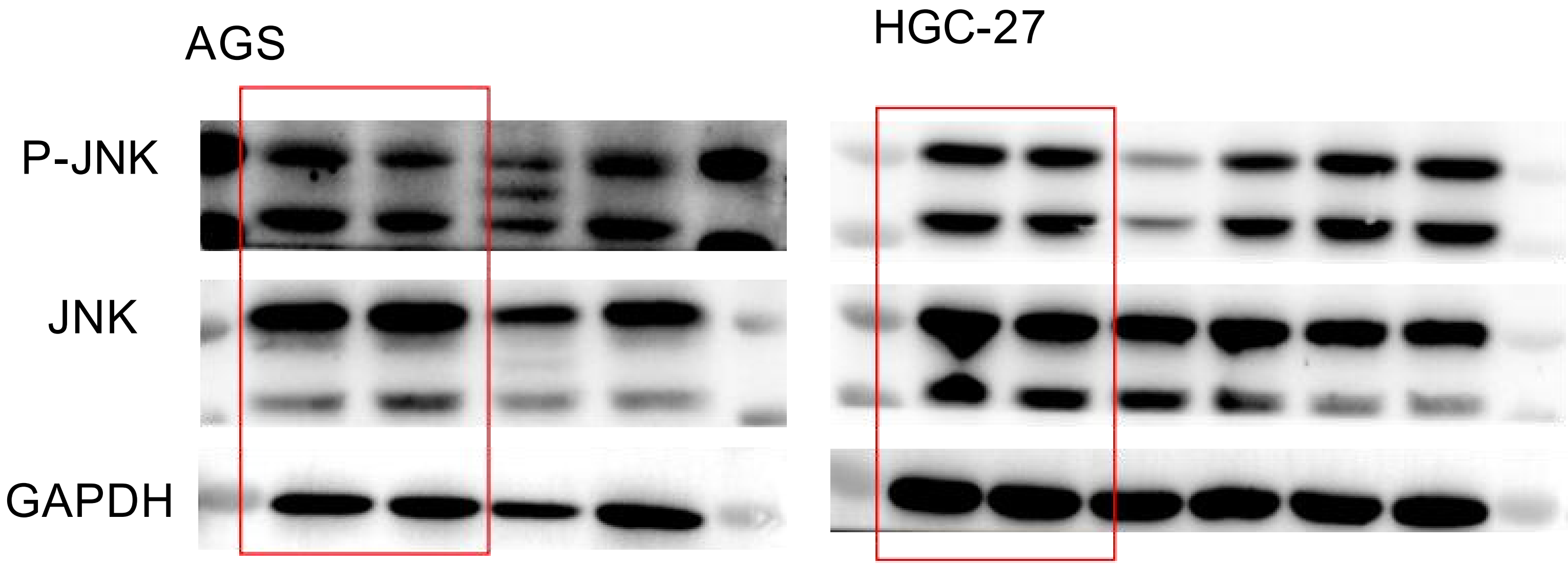

Figure 6D

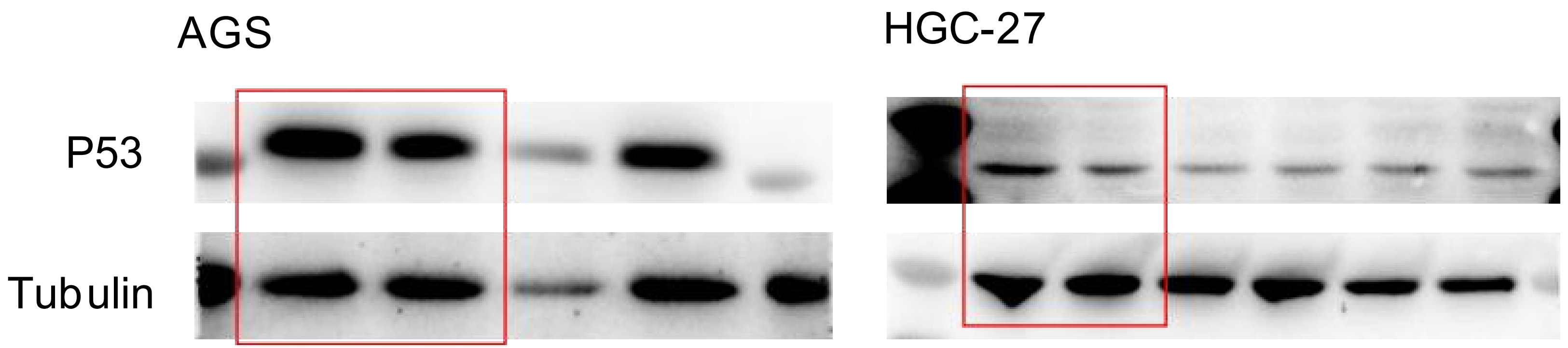

Figure 6I

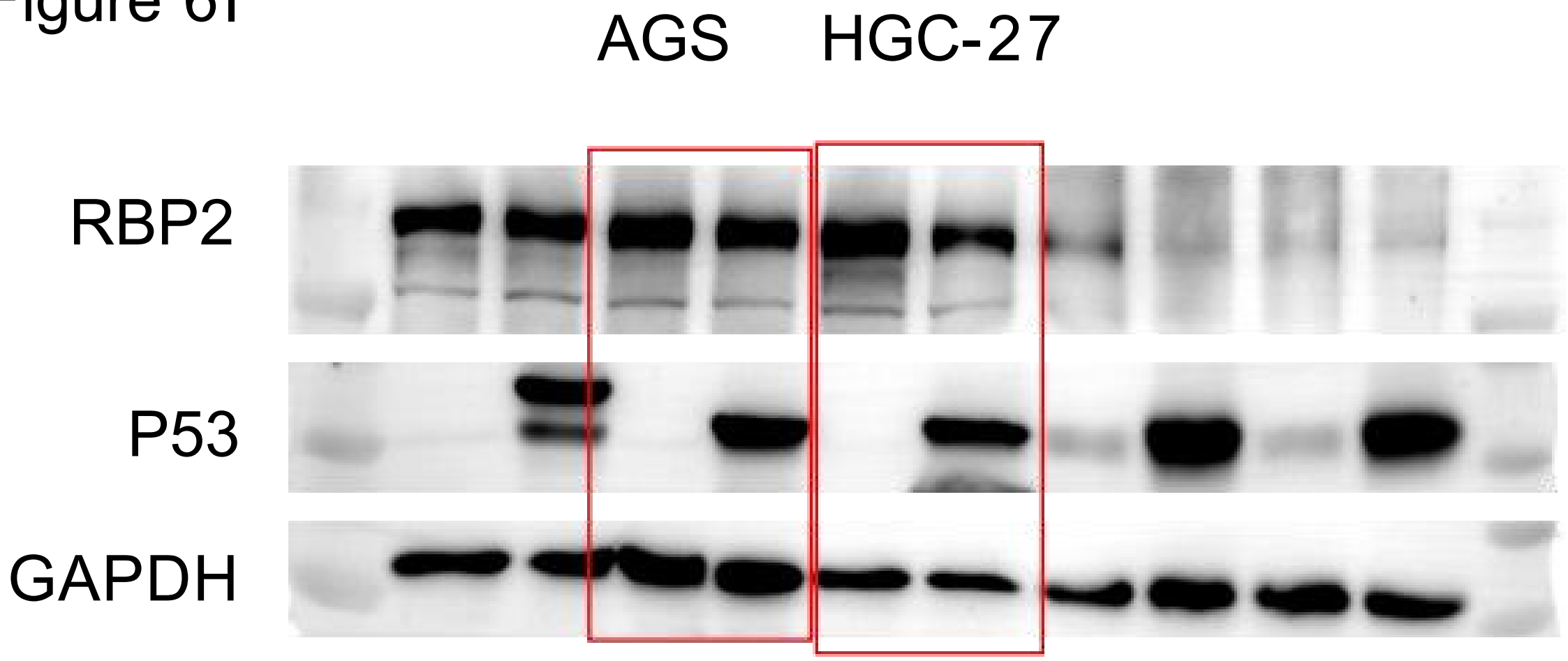

Figure 7A

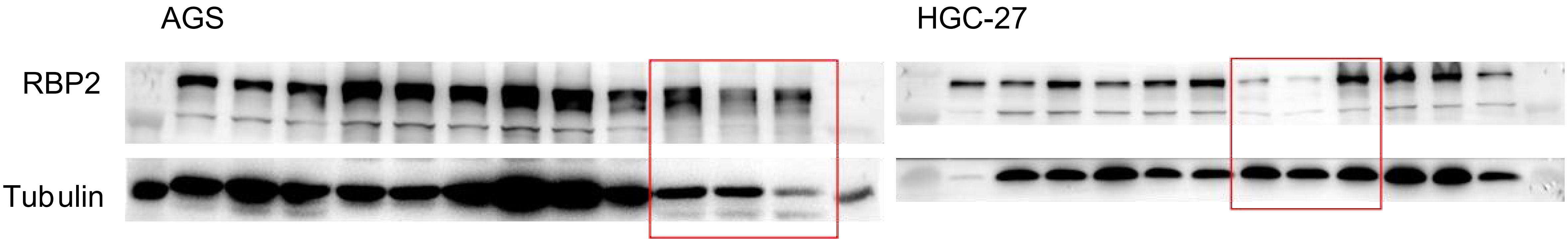

Figure S1B

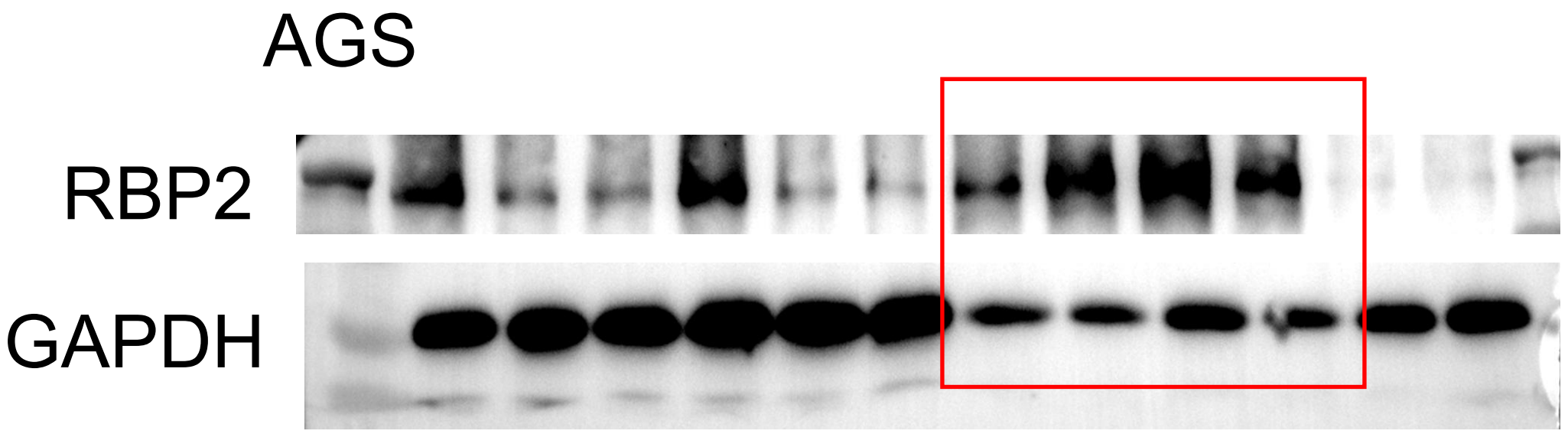

Figure S2A

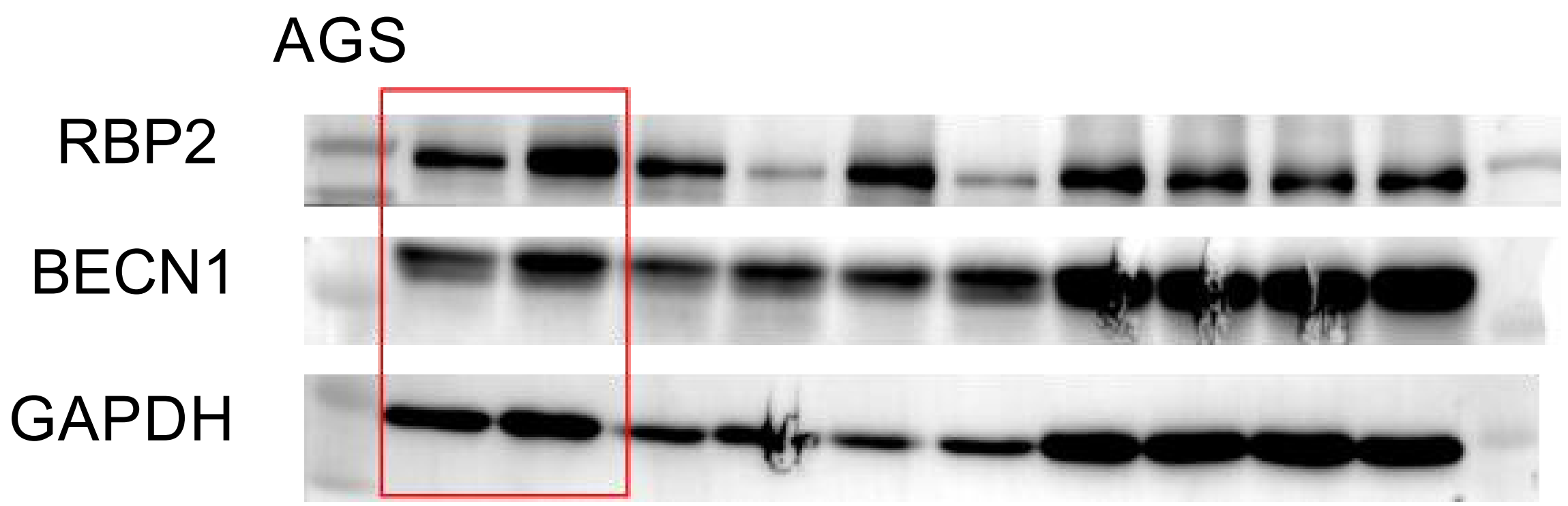

Figure S2C

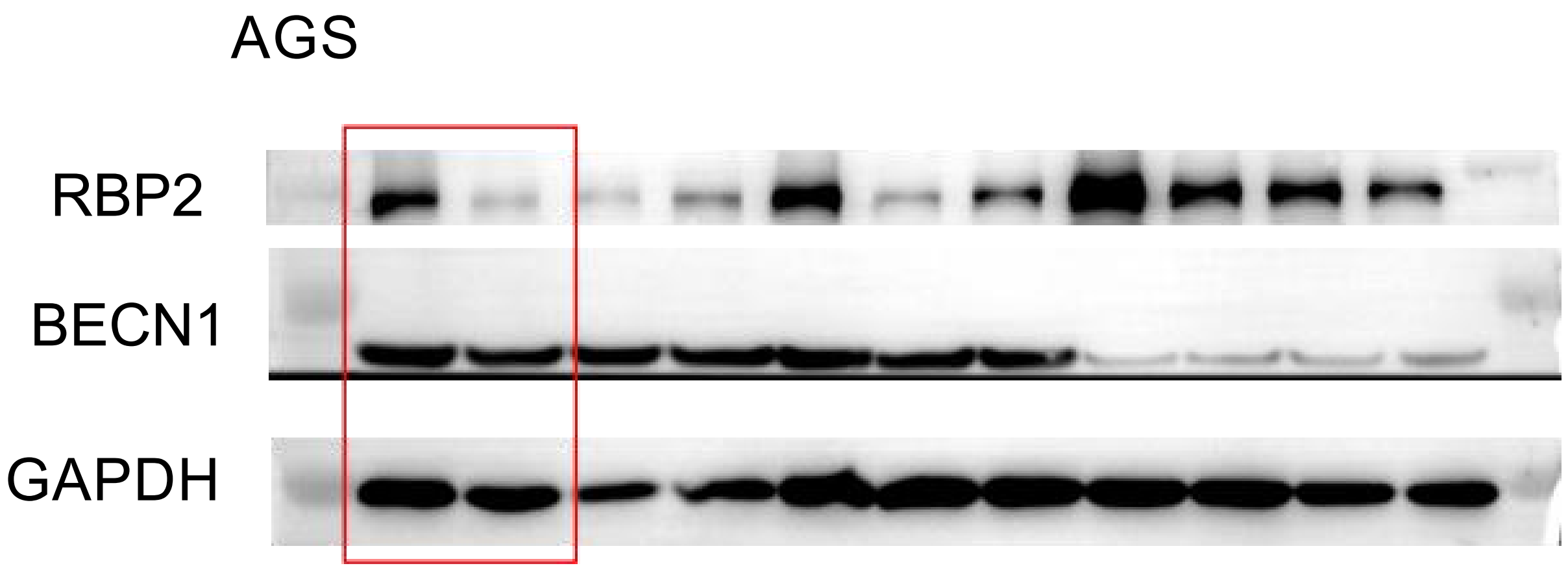

Supplement: Supplementary file 3 [file Supplementaryfile4.pdf]
