# Supplementary material for: The RBP2–BECN1–VEGFA axis orchestrates a self-amplifying circuit to drive malignant progression in gastric carcinoma
Source: Front Oncol. 2026 Sep 7;16:1929764. doi: 10.3389/fonc.2026.1929764 (PMC13590304; doi:10.3389/fonc.2026.1929764)
Supplement: Supplementary file 4 [file Supplementaryfile1.docx]

Supplementary material

**FigureS1**. (A) ChIP-qPCR analysis showing the relative binding levels of RBP2 to motif1, motif2 and motif3 in AGS cells. (B-C) Western blot and RT-qPCR assays validated effective overexpression and knockdown of RBP2 in AGS cells for subsequent functional exploration. Data are mean ± SD. *^ns^P*>0.05, ***P*< 0.01, ****P*<0.001.

**FigureS2.** **RBP2 regulates the expression of BECN1 at protein levels.** (A-B) Western blot analysis of BECN1 protein expression following overexpression of RBP2. (C-D) Western blot analysis of changes in BECN1 protein levels after RBP2 knockdown. Data are mean ± SD.***P*< 0.01.

**FigureS3. STRING-based PPI prediction for RBP2 and VEGFA.**

**Supplementary Table 1**

**The primer sequences for qPCR**

| **Primer** | | **Sequence5’-3’** |
| --- | --- | --- |
| *RBP2* | Forward | GTGCTGGACATCCCCTCAAA |
|  | Reverse | CCACTCATCACAGCCACCAT |
| *BECN1* | Forword | AACCGCAAGATAGTGGCAGA |
|  | Reverse | CTCTCTGATACTGAGCTTCCTCC |
| *VEGF* | Forward | GCAGAATCATCACGAAGTGGT |
|  | Reverse | CCAGGGTCTCGATTGGATGG |
| *P53* | Forward | AGTCACAGCACATGACGGAG |
|  | Reverse | GCCAGACCATCGCTATCTGA |
| *GAPDH* | Forward | GCACCGTCAAGGCTGAGAAC |
|  | Reverse | TGGTGAAGACGCCAGTGGA |
| *MiR-212-3P* | Forward | TTCACCACATAACAGTCTCCAGTC |
|  | Reverse | TATGGTTTTGACGACTGTGTGAT |
| *U6* | Forward | CAGCACATATACTAAAATTGGAACG |
|  | Reverse | ACGAATTTGCGTGTCATCC |
| *BECN1-qdz-1-114bp* | Forward | ACGGGATTTAACCATTTTGGC |
|  | Reverse | AAAGGCCGGGCATGGTG |
| *BECN1*-*qdz-2-107bp* | Forward | GAGACGGGGTTTCACTGTGTT |
|  | Reverse | CGGTGGCTCACGCCT |
| *BECN1*-*qdz-3-98bp* | Forward | CAGGCGTGAGCCACCG |
|  | Reverse | GGTTACCACGGGAAGTGTAGG |

**Supplementary Table 2**

**Homo BECN1 promoter sequence (-1972 - +497)**

**3′-**gcagaatatagttatgtgcaagcactttggaaaatgattcccagtgttaaggaagagacattaaatagctgacacactcttaattctgtagtcccagttatgagtctctatcataagtagccagctcttcattgcaggattattgtaatcacccacaggggaaatagtagaatttccagcggtaaaaaaatacactaaggcagtacatttagtgtagtgtaatgtagccatgataactacaataactgtgtagcaacatagaaaaatgttaaatttaaaaagcagaagcctgggcaacaaagtgagaccccatctcttttgagatggcgtctcgctctgtcaccgaggctggagtgcagtgtgagaccacatctctacaaaaaattttaaaaattagctgggcatggtagtgatcacctgtggtccctgctacactggaggttgaagcaagaggattgcttgagccaggaagtcaaatctgcagtgagccatgtttgtttgttccgcttcactccagcctgggtaacagagtaagacactgtctcaaaataaaaataaaatagacaatactacatacaattttgggttaagcagtggtttctttacaccaaaagcataaacattggactttattgaaatgaaaaactttggccaggcacattggctcacacctgtaatctcagcacttgggaggccacagtgggggattgcaaggggagatgggaaatgttctaaaactggattatggtgatagttgggcaactgtgtaaattactaaaaattattgaactgtacattaaaaagtgtgagtcttatggtatgtaaattataccccataaagttgttttaaaaatgaagtaagtccctctgctcaagacccagtcatctcatctcattcaaagtgaaagccagagctttacaatccctataagagcctaggtggtagctcaacactcttacctccctcaccccatttctgtatctcttttcgttgcccatcttctagccacaccagcctctgctaatccccaaacaggtaccctctgtgctcttgctgttcccttggcctagaatgctcttccttaagatgcaggtaagaattccttcctcaccttcttcaagcttttatttgaatatcactttcttttgttggtttgtgtgtgtgtgtggggggggggggtttgagatggagtttccttctgtcgcccaggctggagtgcagtggcatgatctcgactcactgcaacctccgcctccgggggtcaagcgatttcctaccccagcctcctgagtagctgggattacaggcgcacgccaccatgcccagctaattgtatttttagtagagacgggattaaccatttggccaggctggtctcgaactcctgaccttgtgat**ccgccc**gcctcggcctcccaaagtgctggaattacaagcgtgagccaccatgcccggcctttgttgttgctgttgttgttctgagatggagccttgccctgtcgcccaggctggagtgcagtggccagatctcggctcactgcaacctccacctcccaggttcaagcgattctcctgcctcagcctcccgagtagctgggattaagctgggattataggcgtgccccaccacgcccggctagtttgtattttagtagagacggggtttcactgtgttggccaggctggtctcgaactcctgacctcacgtgat**ccgccc**tcctcggcctccccaagtgctgagattacaggcgtgagccaccgcgcccg**ccgccc**cctgaatttagagaatagcggagcctccccattctctgcggccttggctcctacacttcccgtggtaaccttgttcatccgctgaagcccgctgctttcccagcccggcctctgggggccgctgccgggcctgtgagcctgtggaccaggagctcctgctgccgtcgtagcgtcacgtccggtctcgggcggaagtttt**C**cggcggctaccgggaagtcgctgaagacagagcgatggtagttctggaggcctcgctccggggccgacccgaggccacagtgcctccgcggtagaccggacttgggtgacgggctccgggctcccgaggtgaagagcatcgggggctgaggtgggaccttagaagggagtctgggaaccctcacggctcttattggagtcccttccdgaccctgggctctaaactgcctgctcaggctgtcccggaagcaggtcctccccgtatcataccattcctagagggaacggcgcaggttgggacttccctccctttaccatcgtcaccaaggcatgtggtaaccccgggccggaggtcaagggcgtcgcttctccctaatgttgcctctttccacggcctcagggatggaagggtctaagacgtccaacaacagcaccatgcaggtgagcttcgtgtgccagcgctgcagccagcccctgaaactggacacgagtttcaag **-5′**

RBP2 binding motif

TSS

H3K4me3 peak
